# Integrated computational and experimental evaluation of natural products targeting PGM/AlgC and HisIE in multidrug-resistant respiratory pathogens

**DOI:** 10.3389/fmicb.2026.1891583

**Published:** 2026-08-03

**Authors:** Zhe Wang, Wei Lu, Bing Li, Tianxu Wang, Qiushi Liu, Yunxiang Lv, Kexing Han, Xin Li

**Affiliations:** 1 Department of Respiratory and Critical Care Medicine, The First Affiliated Hospital of Jinzhou Medical, Jinzhou, Liaoning, China; 2 Department of Respiratory and Critical Care Medicine, General Hospital of Northern Theater Command of the Chinese People’s Liberation Army, Shenyang, Liaoning, China; 3 Department of Respiratory and Critical Care Medicine, The First Affiliated Hospital of Bengbu Medical University, Bengbu, Anhui, China

**Keywords:** *Acinetobacter baumannii*, antimicrobial resistance, HisIE, lower respiratory tract infections, molecular docking, molecular dynamics simulation, multidrug-resistant bacteria, natural compounds

## Abstract

**Background:**

The increasing prevalence of multidrug-resistant (MDR) Gram-negative respiratory pathogens has reduced the effectiveness of conventional antimicrobial therapies and created a need for antibacterial strategies targeting bacterial metabolic and virulence-associated functions. AlgC, a phosphomannomutase/phosphoglucomutase involved in cell-envelope and exopolysaccharide biosynthesis, and the bifunctional histidine-biosynthesis enzyme HisIE represent potentially relevant bacterial targets.

**Objective:**

This study evaluated the antibacterial and enzyme-inhibitory activities of three natural products, 5-hydroxysophoranone, 10′-desmethoxystreptonigrin, and isoforsythiaside—and investigated their predicted interactions with AlgC and HisIE using an integrated computational and *in vitro* experimental approach.

**Methods:**

Molecular docking was performed to examine the interactions of the selected compounds with the predicted catalytic or ligand-binding regions of AlgC and HisIE. Two lead protein–ligand complexes were subsequently evaluated using 500-ns molecular dynamics simulations and MM/GBSA analysis. Antibacterial activity against MDR respiratory isolates was assessed using broth dilution and disc-diffusion assays. Cytotoxicity was examined in A549 and BEAS-2B epithelial cells using the MTT assay. Cell-associated antibacterial activity, recombinant-enzyme inhibition, Western blotting, and quantitative real-time PCR were used to assess antibacterial effects and changes in selected metabolic and virulence-associated markers.

**Results:**

The selected natural products showed favorable predicted interactions with the investigated AlgC and HisIE binding regions. Among the tested compounds, 10′-desmethoxystreptonigrin exhibited the strongest predicted interaction with AlgC and showed the lowest minimum inhibitory concentrations, the greatest reduction in cell-associated bacterial burden, and the strongest inhibition of the recombinant enzymes. The two simulated lead complexes retained generally stable protein–ligand interactions during the molecular dynamics trajectories. Treatment with the compounds was also associated with reduced expression of selected metabolic and virulence-associated markers, including pgm/algC, hisIE, lasR, pslA, and ompA. Isoforsythiaside demonstrated comparatively greater epithelial-cell compatibility, whereas 10′-desmethoxystreptonigrin showed greater antibacterial potency but lower host-cell tolerance.

**Conclusion:**

The combined computational and experimental findings identify 10′-desmethoxystreptonigrin and 5-hydroxysophoranone as candidate natural-product scaffolds with antibacterial and recombinant AlgC/HisIE-inhibitory activities. The observed enzyme inhibition and changes in selected molecular markers are consistent with possible interference in AlgC- and HisIE-associated processes; however, they do not by themselves establish direct intracellular target engagement or pathway-specific causality. Further studies using target-engagement assays, genetically defined bacterial strains, broader clinical-isolate panels, biofilm and quorum-sensing assays, and *in vivo* respiratory-infection models are required to validate the proposed mechanism, safety, and translational potential.

## Highlights

- Three structurally distinct natural products were evaluated against AlgC and HisIE.
- 10′-Desmethoxystreptonigrin showed the strongest overall antibacterial and recombinant-enzyme-inhibitory activity.
- Two lead protein–ligand complexes retained generally stable interactions during molecular dynamics simulations.
- Treatment reduced the expression of selected metabolic and virulence-associated markers.
- Isoforsythiaside showed greater epithelial-cell compatibility but lower antibacterial potency.

## Introduction

Lower respiratory tract infections (LRTIs) remain a major cause of morbidity and mortality worldwide, particularly among hospitalized and critically ill patients (GBD 2016 Lower Respiratory Infections Collaborators, 2018; Torres et al., 2019). The growing prevalence of multidrug-resistant (MDR) Gram-negative pathogens, including *Acinetobacter baumannii*, *Pseudomonas aeruginosa*, and *Klebsiella pneumoniae*, has substantially complicated the management of hospital-acquired and ventilator-associated pneumonia (Bassetti et al., 2018; Tacconelli et al., 2018). These pathogens employ multiple resistance mechanisms, including efflux pump activation, enzymatic antibiotic degradation, outer membrane remodeling, and biofilm formation, all of which contribute to reduced antibiotic susceptibility and persistent respiratory infections (Pang et al., 2019; Rajput et al., 2024). In addition to conventional resistance determinants, recent studies have highlighted the importance of metabolic adaptation and stress-response pathways in promoting bacterial persistence within the lung microenvironment (Crabbé et al., 2019; Meylan et al., 2017).

Phosphoglucomutase/phosphomannomutase (PGM/AlgC) has emerged as a metabolically important antibacterial target because of its involvement in carbohydrate metabolism, lipopolysaccharide biosynthesis, and exopolysaccharide production (Rachmawati et al., 2022; Vassoler Serrato, 2024). This enzyme exhibits phosphomannomutase/phosphoglucomutase activity and catalyzes the reversible interconversion of mannose-1-phosphate and mannose-6-phosphate, as well as glucose-1-phosphate and glucose-6-phosphate (Regni et al., 2000). Structural characterization of *P. aeruginosa* PGM has demonstrated a conserved catalytic cleft containing a phosphoserine-centered active site that facilitates phosphoryl transfer reactions in the presence of divalent metal ions (Regni et al., 2006; Rehman and Rehm, 2013). Functional studies have further shown that perturbation of PGM-associated pathways may impair membrane stability, biofilm development, and antibiotic tolerance in Gram-negative pathogens (Ashniev et al., 2022; Meylan et al., 2017).

Another potentially relevant but comparatively underexplored bacterial enzyme is the bifunctional histidine-biosynthesis enzyme HisIE. *De novo* histidine biosynthesis is absent in mammalian cells and may support bacterial survival under nutrient-limited conditions (Ingle, 2011). HisIE catalyzes early steps of the histidine biosynthetic pathway and contains conserved catalytic regions that are suitable for small-molecule inhibition (Mascher et al., 2006). Previous reports have linked histidine biosynthesis to oxidative stress regulation, metabolic homeostasis, and bacterial adaptation during host colonization (Bender, 2012; Cushnie et al., 2014). Consequently, inhibition of HisIE could interfere with bacterial metabolic adaptation under nutrient-limited or stress-associated conditions.

Combined investigation of AlgC and HisIE may provide a complementary framework for examining bacterial metabolic adaptation and virulence-associated processes. Natural products represent an important source of structurally diverse bioactive compounds with multitarget therapeutic potential (Atanasov et al., 2021). Therefore, the compounds selected in this study were chosen to represent structurally diverse natural-product scaffolds with functional groups capable of interacting with bacterial catalytic pockets and with reported relevance to anti-infective or enzyme-modulatory activity (Andima et al., 2022; Chepkirui et al., 2021). The selected compounds contain hydroxyl, quinone, or glycosidic groups that may contribute to their predicted interactions with the investigated protein-binding regions (Andima et al., 2022). Several natural compounds have previously demonstrated antimicrobial, antioxidant, and enzyme-modulatory activities, supporting their potential use in structure-based antibacterial screening approaches (Cushnie et al., 2014; Newman and Cragg, 2020).

Recent advances in computational drug-discovery methods, including molecular docking, molecular dynamics (MD) simulations, and MM/GBSA free-energy analysis, have improved the prediction of ligand–target interactions at atomic resolution (Wang et al., 2019; Yan et al., 2025). Integration of computational predictions with antibacterial, cytotoxicity, enzyme-inhibition, and molecular-expression assays may support the preliminary prioritization of candidate compounds.

10′-Desmethoxystreptonigrin is a streptonigrin analog originally isolated from Streptomyces albus and reported as an inhibitor identified during screening for farnesylation of Ras p21 protein. Importantly, this compound has been reported to show marked cytotoxic activity against several human tumor cell lines and broad-spectrum antibacterial activity, supporting its pharmacological relevance as a bioactive microbial natural product. Streptonigrin and related aminoquinone alkaloids are also recognized for diverse biological activities, including antibacterial and antitumor effects, although toxicity remains an important consideration during lead optimization. Therefore, inclusion of 10′-Desmethoxystreptonigrin in the present study is pharmacologically justified because it represents a structurally complex natural-product scaffold with previously reported antibacterial potential and possible relevance for antimicrobial lead discovery (Liu et al., 1992; Nabihah Nasir et al., 2023).

Despite increasing interest in metabolic pathway inhibition as an antibacterial strategy, limited studies have examined simultaneous targeting of carbohydrate metabolism and amino-acid biosynthesis pathways in MDR respiratory pathogens. In particular, the combined inhibition of PGM/AlgC and HisIE may provide a complementary approach by interfering with cell-envelope biosynthesis, adaptive metabolism, stress tolerance, and virulence-associated signaling. However, the potential of natural compounds to act as dual-target modulators of these enzymes remains insufficiently explored.

Accordingly, the present study evaluated the predicted and recombinant-enzyme inhibitory interactions of 5-Hydroxysophoranone, 10′-Desmethoxystreptonigrin, and Isoforsythiaside against PGM/AlgC and HisIE using an integrated computational and experimental framework. The study further evaluated antibacterial activity, cytocompatibility, cell-associated bacterial reduction, enzyme inhibition, and modulation of metabolic and virulence-associated markers in representative MDR respiratory pathogens. The study examined whether the antibacterial effects of selected natural products were accompanied by inhibition of recombinant AlgC and HisIE and changes in selected metabolic and virulence-associated markers.

## Materials and methods

### Experimental design and reproducibility

The study was designed as an integrated computational and *in vitro* experimental investigation to evaluate the dual-target inhibitory potential of selected natural compounds against PGM/AlgC and HisIE. Computational analyses included molecular docking, molecular dynamics simulation, and MM/GBSA binding free-energy estimation. Experimental validation included antibacterial susceptibility testing, epithelial-cell cytotoxicity assessment, cell-associated antibacterial activity, enzyme inhibition assays, Western blotting, and qPCR analysis. Unless otherwise stated, all *in vitro* experiments were performed using at least three independent biological replicates, and each assay included untreated control, vehicle control, and positive-control groups where appropriate. Data acquisition and analysis procedures were standardized across treatment groups to ensure reproducibility.

### Chemicals, compounds, microbial strains, and cell lines

All analytical-grade chemicals, culture media, electrophoresis reagents, dimethyl sulfoxide (DMSO), MTT reagent, phosphate-buffered saline (PBS), Triton X-100, bovine serum albumin, and related laboratory reagents were obtained from commercial suppliers including Sigma-Aldrich (St. Louis, MO, United States). The selected compounds, 5-Hydroxysophoranone, 10′-Desmethoxystreptonigrin, and Isoforsythiaside were procured at ≥95% purity and prepared as 10 mM stock solutions in sterile DMSO.

Human alveolar epithelial A549 cells (ATCC CCL-185) and bronchial epithelial BEAS-2B cells (ATCC CRL-3588) were used for cytotoxicity and infection assays. Reference strains included Acinetobacter baumannii ATCC 19606 and Pseudomonas aeruginosa ATCC 27853, while multidrug-resistant respiratory clinical isolates included representative Acinetobacter baumannii, Pseudomonas aeruginosa, and Enterobacterales isolates recovered from archived, de-identified lower respiratory tract specimens. All microbiological and mammalian cell culture procedures were conducted under Biosafety Level-2 conditions following institutional biosafety guidelines.

### Microbial strain source, identification, and antimicrobial-resistance confirmation

The bacterial strains used in this study included reference strains and multidrug-resistant respiratory clinical isolates. Reference strains, including *Acinetobacter baumannii* ATCC 19606 and *Pseudomonas aeruginosa* ATCC 27853, were obtained from the American Type Culture Collection and used as quality-control/reference organisms. The multidrug-resistant respiratory isolates were obtained from archived, de-identified lower respiratory tract specimens collected during routine clinical microbiology diagnostics, including sputum, bronchoalveolar lavage, and tracheal aspirate samples. No additional patient sampling was performed specifically for this study.

All clinical isolates were initially identified using standard microbiological culture characteristics and biochemical testing, followed by confirmation using an automated microbial identification system or MALDI-TOF mass spectrometry where available. Antimicrobial susceptibility profiles were determined using standard broth microdilution, automated susceptibility testing, or disc diffusion methods according to recognized clinical microbiology guidelines. Isolates were classified as multidrug-resistant when they showed non-susceptibility to at least one agent in three or more antimicrobial categories. Only confirmed MDR respiratory isolates were included in the experimental analysis. All isolates were stored at −80 °C in glycerol-containing medium and subcultured on appropriate agar medium before experimental use. All microbiological procedures were performed under Biosafety Level-2 conditions.

Because the clinical isolates were archived and de-identified bacterial isolates obtained from routine diagnostic workflows, no personally identifiable patient information was used in this study. The use of these anonymized isolates was conducted in accordance with institutional biosafety and ethical requirements.

### Selection criteria for the natural compounds

The selected compounds 5-Hydroxysophoranone, 10′-Desmethoxystreptonigrin, and Isoforsythiaside were selected based on predefined criteria relevant to natural-product-based antibacterial discovery. First, compounds were prioritized if they belonged to natural-product classes with previously reported anti-infective, antimicrobial, or enzyme-modulatory potential. Second, structurally diverse compounds containing hydroxyl, quinone, or glycosidic functional groups were selected because these moieties may support hydrogen bonding, electrostatic interactions, and catalytic-pocket accommodation within bacterial enzymes. Third, compounds were chosen to represent different chemical scaffolds, allowing comparison of binding behavior and biological activity across structurally distinct phytochemicals. Fourth, only compounds with available structural information, acceptable purity, and suitability for downstream *in vitro* testing were included. Based on these criteria, the selected compounds were evaluated against PGM/AlgC and HisIE using molecular docking, molecular dynamics simulation, and experimental validation assays. This selection strategy is consistent with previous reports highlighting the importance of structurally diverse natural products, microbial natural-product scaffolds, and flavonoid-related compounds in anti-infective and antibacterial drug-discovery studies (Atanasov et al., 2021; Liu et al., 1992; Nabihah Nasir et al., 2023; Nasim et al., 2022; Newman and Cragg, 2020; Shamsudin et al., 2022; Thebti et al., 2023).

### Ligand library source and reference compound

The focused ligand library used for molecular docking consisted of three selected natural compounds, namely 5-Hydroxysophoranone, 10′-Desmethoxystreptonigrin, and Isoforsythiaside. The ligand structures were retrieved from the PubChem compound database using their corresponding PubChem compound identifiers. PubChem was used as the primary source for two-dimensional structures, canonical chemical information, and structure-data files. The retrieved ligand structures were converted into three-dimensional conformations, protonated at physiological pH, and energy-minimized before docking. Meropenem was included as the reference antibacterial comparator because it is a clinically used broad-spectrum carbapenem antibiotic commonly applied against serious Gram-negative bacterial infections. The complete list of screened ligands, their database source, PubChem CID, chemical class, and role in the study is provided in Supplementary Table 8.

PubChem entries identify 5-Hydroxysophoranone/Hydroxysophoranone as CID 42607927, 10′-Desmethoxystreptonigrin as CID 131994, Isoforsythiaside as CID 23958169, and Meropenem as CID 441130.

### Cell culture and maintenance

A549 and BEAS-2B cells were maintained in DMEM/F12-based complete culture medium supplemented with 10% heat-inactivated fetal bovine serum, 1% penicillin–streptomycin, and 1% L-glutamine. Cells were incubated at 37 °C in a humidified atmosphere containing 5% CO_2_ and passaged at approximately 70–80% confluence. Prior to infection experiments, cells were cultured in antibiotic-free medium and seeded in appropriate culture plates according to assay requirements.

For infection assays, epithelial monolayers were infected with bacterial suspensions at an optimized multiplicity of infection (MOI) of 10. Following incubation, extracellular bacteria were removed by washing with sterile PBS, and compound treatments were applied at concentrations derived from preliminary antibacterial screening assays.

### Receptor retrieval

The three-dimensional receptor structures used for molecular docking were retrieved from the RCSB Protein Data Bank after screening available experimentally resolved bacterial structures for biological relevance, crystallographic quality, active-site information, and suitability for structure-based ligand docking. The PGM/AlgC receptor was selected as the crystal structure of phosphomannomutase/phosphoglucomutase from Pseudomonas aeruginosa (PDB ID: 1K35). This structure was chosen because PGM/AlgC is directly involved in alginate and lipopolysaccharide biosynthesis, both of which are associated with P. aeruginosa virulence, biofilm formation, and respiratory infection persistence. The 1K35 structure was solved by X-ray diffraction at 2.20 Å resolution and provides a well-defined active-site cleft suitable for structure-based inhibitor screening. The reported structural information indicates that residues from all four domains contribute to the central active-site cleft, supporting its relevance for catalytic-pocket docking and inhibitor-design studies.

The HisIE receptor was selected as the crystal structure of the bifunctional histidine biosynthesis enzyme from Shigella flexneri (PDB ID: 6J2L). This structure was chosen because HisIE catalyzes early steps in the bacterial histidine biosynthesis pathway, which is absent in animals and conserved among Gram-negative bacteria, making it a relevant antibacterial target. The 6J2L structure was solved by X-ray diffraction at 2.17 Å resolution and contains structurally characterized HisE and HisI functional regions with conserved residues located in putative ligand-binding grooves. Therefore, 6J2L was considered suitable for identifying ligand-binding interactions within biologically relevant catalytic/binding regions. Overall, 1K35 and 6J2L were selected because they are experimentally resolved, biologically relevant to Gram-negative bacterial metabolism and virulence, and provide structurally defined binding regions appropriate for molecular docking analysis.

### Molecular docking analysis

Before molecular docking, the retrieved PGM/AlgC and HisIE protein structures were prepared and energy-minimized. Protein preparation included removal of non-essential crystallographic water molecules, addition of polar hydrogens, assignment of Kollman charges, merging of non-polar hydrogens, and correction of unfavorable local contacts. Energy minimization was performed using the Schrödinger Protein Preparation Wizard with the OPLS4 force field prior to docking to improve local geometry and remove steric clashes while preserving the experimentally resolved receptor conformation. The minimized receptor structures were converted into PDBQT format using AutoDockTools 1.5.7/MGLTools and visually inspected using Discovery Studio Visualizer before docking analysis. Ligand structures of 5-Hydroxysophoranone, 10′-Desmethoxystreptonigrin, Isoforsythiaside, and Meropenem were prepared before docking. The two-dimensional ligand structures were converted into three-dimensional conformations, protonated at physiological pH, and geometry-optimized using Avogadro software with the MMFF94 force field. The energy-minimized ligand structures were subsequently converted into PDBQT format using AutoDockTools 1.5.7/MGLTools for AutoDock Vina docking.

Docking simulations were performed using AutoDock Vina following structure-based virtual screening principles (Lionta et al., 2014). Before grid generation, the active-site/binding-pocket regions of PGM/AlgC and HisIE were identified using a combined structure-guided approach. First, catalytic-site information was obtained from the Protein Data Bank records and published structural studies of PGM/AlgC and HisIE. Second, the prepared receptor structures were analyzed using CASTp 3.0 to identify surface-accessible pockets, pocket volume, pocket area, and residues lining the predicted cavities. Third, the predicted pockets were visually inspected in Discovery Studio Visualizer to confirm that the selected cavities corresponded to conserved catalytic or putative ligand-binding regions rather than nonspecific surface depressions. For PGM/AlgC, the selected docking pocket corresponded to the central catalytic cleft formed by residues from multiple domains. For HisIE, the selected pocket corresponded to the conserved putative ligand-binding groove within the HisE/HisI functional region. The final docking grids were centered on the identified catalytic/binding pockets and adjusted to fully cover the pocket and adjacent ligand-accessible residues. The grid-box center coordinates and dimensions used for each receptor are reported in Supplementary Table 6. Grid center coordinates were defined from the centroid of the selected catalytic/binding pocket, while grid dimensions were selected to cover the full pocket volume and adjacent ligand-accessible residues. Docking was therefore restricted to biologically relevant binding regions rather than performed across the entire protein surface. Docking simulations were performed using a consistent AutoDock Vina exhaustiveness value of 32 for all receptor–ligand docking runs to improve conformational search depth and ensure comparable docking conditions across PGM/AlgC and HisIE. For each ligand–target pair, at least 20 binding poses were generated, and the best-ranked pose was selected based on docking score, catalytic-site occupancy, hydrogen-bonding pattern, hydrophobic interactions, electrostatic complementarity, and absence of major steric clashes. The active-site identification approach, selected docking regions, and grid-box parameters are summarized in Supplementary Table 6.

### Docking protocol validation

The selected receptor structures were examined for the presence of co-crystallized organic ligands suitable for redocking-based validation. The PGM/AlgC structure 1K35 and the HisIE structure 6J2L did not contain suitable co-crystallized organic inhibitor or substrate ligands that could be removed and redocked for conventional RMSD-based validation. Therefore, self-redocking and RMSD calculation using native organic ligands could not be performed for the selected receptor structures. Redocking of metal ions was not considered appropriate because it would not validate the docking behavior of flexible organic ligands.

To support docking reliability, active-site selection was performed using published structural information, PDB-reported catalytic/binding-site features, CASTp 3.0 pocket prediction, and Discovery Studio visual inspection. Docking grids were restricted to the identified catalytic/binding pockets rather than the entire protein surface. Docking poses were further evaluated based on docking score, catalytic-pocket occupancy, hydrogen-bonding pattern, hydrophobic and electrostatic interactions, absence of major steric clashes, and consistency with conserved binding-site residues. In addition, the stability of the lead docked complexes was further examined using 500 ns molecular dynamics simulations and MM/GBSA analysis.

### Molecular dynamics simulation and MM/GBSA analysis

Lead protein–ligand complexes identified from molecular docking were subjected to molecular dynamics (MD) simulations using the Desmond module of Schrödinger software (Hollingsworth and Dror, 2018; Wang et al., 2019; Yan et al., 2025). The selected lead complexes included 10′-Desmethoxystreptonigrin–PGM/AlgC and 5-Hydroxysophoranone–HisIE. Each system was prepared using the System Builder module and parameterized using the OPLS4 force field. The protein–ligand complexes were solvated in a system-specific orthorhombic TIP3P water box generated with a minimum 10 Å buffer distance between the protein–ligand complex and the simulation box boundary. Because the box size was generated automatically according to the dimensions of each prepared protein–ligand complex, fixed manual box dimensions were not assigned. The systems were neutralized with appropriate counterions, and 0.15 M NaCl was added to approximate physiological ionic strength.

Before production simulation, each system was subjected to energy minimization and equilibration using the Desmond default relaxation protocol. The relaxation procedure included restrained energy minimization, short restrained simulations under NVT and NPT conditions, and final unrestrained equilibration before production MD. Production simulations were performed for 500 ns under the NPT ensemble at 300 K and 1 atm pressure. Temperature was controlled using a Nosé–Hoover thermostat, and pressure was maintained using a Martyna–Tobias–Klein barostat. Trajectory frames were recorded at 100 ps intervals for subsequent analysis.

Trajectory analysis was performed using the Desmond Simulation Interaction Diagram and associated trajectory-analysis tools. Protein RMSD was calculated after alignment to the initial protein backbone, while ligand RMSD was calculated using ligand heavy atoms after fitting to the receptor. RMSF was calculated on a residue-wise basis to evaluate local flexibility. Additional analyses included protein secondary-structure elements, protein–ligand contact mapping, hydrogen-bond interactions, hydrophobic contacts, ionic interactions, water bridges, radius of gyration, molecular surface area, solvent-accessible surface area, polar surface area, ligand RMSF, and ligand torsional profiles. Binding free energies were estimated using Prime MM/GBSA from representative equilibrated trajectory frames. The calculated energy components included Coulombic, hydrogen-bonding, lipophilic, and solvation energy contributions.

The complete MD simulation parameters for both lead protein–ligand complexes, including software, force field, water model, box construction, ion concentration, equilibration protocol, thermostat/barostat settings, simulation length, trajectory-recording interval, and trajectory-analysis outputs, are summarized in Supplementary Table 9.

### Computational reproducibility statement

All computational procedures were documented to improve reproducibility. Receptor structures were retrieved from the RCSB Protein Data Bank, and ligand structures were obtained from PubChem. Receptors were prepared by removing non-essential crystallographic water molecules, adding polar hydrogens, assigning Kollman charges, merging non-polar hydrogens, correcting unfavorable local contacts, and converting the prepared structures into PDBQT format using AutoDockTools 1.5.7/MGLTools. Ligands were converted into three-dimensional conformations, protonated at physiological pH, geometry-optimized using Avogadro with the MMFF94 force field, and converted into PDBQT format using AutoDockTools 1.5.7/MGLTools. Active-site selection was performed using PDB-reported structural information, published catalytic-site evidence, CASTp 3.0 pocket prediction, and Discovery Studio Visualizer inspection. Molecular docking was performed using AutoDock Vina, and docking poses were evaluated based on docking score, catalytic-pocket occupancy, hydrogen-bonding pattern, hydrophobic interactions, electrostatic complementarity, and absence of major steric clashes. Molecular dynamics simulations were performed using Desmond with the OPLS4 force field, TIP3P water model, orthorhombic water box, 10 Å buffer distance, 0.15 M NaCl, NPT ensemble, 300 K, 1 atm pressure, 500 ns production simulation, and 100 ps trajectory-recording interval. The computational parameters used for receptor preparation, ligand preparation, docking, interaction analysis, molecular dynamics simulation, and MM/GBSA analysis are summarized in Supplementary Table 10.

### MTT cytotoxicity assay

Cytotoxicity of the selected compounds was assessed using the MTT assay in A549 and BEAS-2B cells. Cells were seeded in 96-well plates at a density of approximately 8 × 10^3^ cells/well and incubated overnight. Following attachment, cells were exposed to serial concentrations of the compounds for 48 h.

After treatment, MTT reagent was added to a final concentration of 0.5 mg/mL and incubated for 3–4 h. Formazan crystals were dissolved using DMSO, and absorbance was measured at 570 nm with background correction at 630–690 nm. Cell viability was calculated relative to untreated controls, and CC50 values were determined using nonlinear regression analysis.

### Antibacterial susceptibility testing

Antibacterial activity was evaluated using broth macrodilution and agar disc diffusion assays, consistent with commonly used *in vitro* screening approaches for natural-product antibacterial evaluation (Shamsudin et al., 2022; Thebti et al., 2023). Bacterial suspensions were adjusted to 0.5 McFarland standard and diluted to the required inoculum density.

Minimum inhibitory concentration (MIC) and minimum bactericidal concentration (MBC) values were determined in Mueller–Hinton broth following incubation with serial compound dilutions. Meropenem was used as the reference antibiotic comparator. For disc diffusion assays, sterile discs impregnated with fixed concentrations of compounds were placed onto inoculated agar plates and incubated for 18–24 h prior to measurement of inhibition-zone diameters.

Selectivity index (SI) values were calculated using the following relationship:


$$S\,I=\frac{C\,C\,50}{M\,I\,C}$$

where CC50 represents the 50% cytotoxic concentration and MIC represents the minimum inhibitory concentration.

### Cell-associated antibacterial activity assay

The cell-associated antibacterial activity of the compounds was evaluated in infected A549 epithelial cells. Following bacterial infection and compound treatment, monolayers were washed with PBS and lysed using 0.1% Triton X-100. Serial dilutions of the lysates were plated on agar plates for enumeration of surviving cell-associated bacteria as colony-forming units (CFU/mL).

Parallel wells were used for assessment of epithelial viability to ensure that antibacterial effects were not attributable to nonspecific host-cell toxicity.

### Enzyme inhibition assay

Recombinant PGM/AlgC and HisIE enzymes were used to evaluate direct target inhibition by the selected compounds. Serial compound concentrations were incubated with purified recombinant enzymes under optimized reaction conditions.

PGM/AlgC activity was measured using a coupled enzymatic assay based on NADPH generation monitored spectrophotometrically at 340 nm. HisIE activity was evaluated using a phosphate-release assay compatible with phosphoribosyl-ATP turnover detection. IC50 values were determined by nonlinear regression analysis of concentration–response curves.

### Western blot analysis

Protein-expression analysis was performed to assess modulation of metabolic and virulence-associated bacterial proteins following compound treatment. Bacterial pellets were lysed using RIPA buffer supplemented with protease inhibitors and subjected to probe sonication. Protein concentrations were quantified using the Pierce BCA Protein Assay Kit (Thermo Fisher Scientific, United States).

Equal amounts of total protein (20–30 μg) were separated by SDS-PAGE and transferred onto PVDF membranes. Membranes were blocked in 5% skim milk and incubated overnight at 4 °C with primary antibodies against PGM/AlgC, HisIE, OmpA, LasR, PslA, and OprL. GroEL was used as the housekeeping loading control.

Following incubation with horseradish peroxidase-conjugated secondary antibodies, protein bands were visualized using enhanced chemiluminescence substrate. Band intensities from three independent Western blot experiments were quantified using ImageJ software. For each target protein, background-corrected band intensity was normalized to the corresponding GroEL loading-control band and expressed as fold change relative to the untreated control group. Quantified densitometric data are presented as mean ± SD from three independent experiments.

### Quantitative real-time PCR (qPCR) analysis

Total RNA was extracted from treated and untreated bacterial cultures using the Qiagen RNeasy Mini Kit with on-column DNase treatment. Complementary DNA synthesis was performed using reverse-transcription master mix according to the manufacturer’s instructions.

Quantitative PCR amplification was performed using SYBR Green chemistry under standard cycling conditions consisting of initial denaturation at 95 °C for 5 min followed by 40 amplification cycles. Relative gene-expression levels were calculated using the 2^–^ΔΔCt method with *rpoB* and 16S rRNA used as housekeeping reference genes.

The analyzed gene panel included *pgm/algC*, *hisIE*, *ompA*, *lasR*, *pslA*, *algD*, *katG*, and *dnaK*. Primer sequences used for qPCR amplification are provided in Supplementary Table 3.

### Statistical analysis

All experiments were performed using at least three independent biological replicates. Data are presented as mean ± standard deviation (SD). Statistical analyses were conducted using GraphPad Prism software (GraphPad Software Inc., San Diego, CA, United States).

Comparisons between multiple groups were performed using one-way or two-way analysis of variance (ANOVA) followed by Tukey’s or Dunnett’s *post-hoc* test where appropriate. Differences were considered statistically significant at *p* < 0.05.

## Results

### Molecular docking analysis of selected compounds against PGM/AlgC and HisIE

**FIGURE 1:**
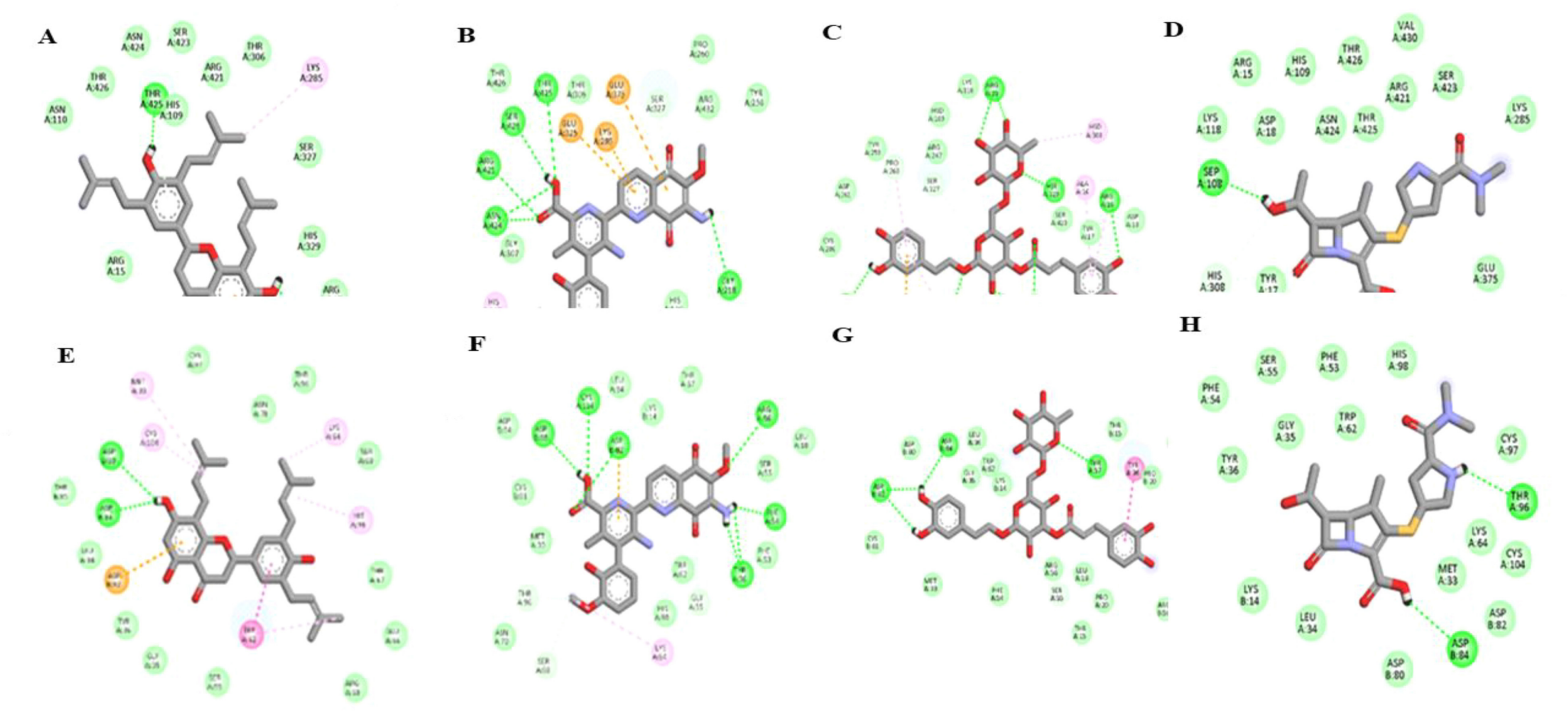
Molecular docking interaction profiles of selected compounds within the active sites of PGM/AlgC and HisIE enzymes. **(A)** 5-Hydroxysophoranone–PGM/AlgC complex, **(B)** 10′-Desmethoxystreptonigrin–PGM/AlgC complex, **(C)** Isoforsythiaside–PGM/AlgC complex, **(D)** Meropenem–PGM/AlgC complex, **(E)** 5-Hydroxysophoranone–HisIE complex, **(F)** 10′-Desmethoxystreptonigrin–HisIE complex, **(G)** Isoforsythiaside–HisIE complex, and **(H)** Meropenem–HisIE complex. Hydrogen-bond interactions are indicated by green dashed lines, while hydrophobic, electrostatic, π-related, and van der Waals interactions are represented by additional colored markers. The selected compounds demonstrated interactions with key catalytic residues within the active regions of PGM/AlgC and HisIE enzymes.

The three-dimensional receptor structures and identified catalytic/binding regions of PGM/AlgC and HisIE are shown in Figure 1. PGM/AlgC displayed a central catalytic cleft suitable for ligand accommodation, whereas HisIE exhibited a conserved ligand-binding groove within the functionally relevant HisE/HisI region. These structurally defined pockets were used for docking-grid placement and provided the basis for evaluating whether the selected natural compounds could interact with biologically relevant enzyme regions rather than nonspecific surface sites.

Molecular docking analysis demonstrated favorable interactions of the selected compounds with the catalytic/binding regions of both PGM/AlgC and HisIE enzymes (Table 1, Figure 1, and Supplementary Figure 6). Among the tested compounds, 10′-Desmethoxystreptonigrin exhibited the strongest predicted affinity toward PGM/AlgC, with a docking score of −10.000 kcal/mol, exceeding that of the reference antibiotic Meropenem (−6.682 kcal/mol). This stronger predicted interaction is biologically relevant because PGM/AlgC contributes to carbohydrate metabolism, lipopolysaccharide biosynthesis, and alginate-associated virulence. Therefore, stable ligand interaction within the PGM/AlgC catalytic cleft suggests a possible mechanism by which 10′-Desmethoxystreptonigrin may interfere with bacterial cell-envelope biosynthesis, biofilm-associated adaptation, and metabolic persistence.

**TABLE 1:** Molecular docking scores of selected compounds against PGM/AlgC and HisIE enzymes.

Compound	PGM/AlgC docking score (kcal/mol)	HisIE docking score (kcal/mol)
5-Hydroxysophoranone	−7.961	−8.800
10′-Desmethoxystreptonigrin	**−10.000**	−7.961
Isoforsythiaside	−7.137	−7.081
Meropenem	−6.682	−6.812

Values represent binding affinities predicted by AutoDock Vina. More negative values indicate stronger predicted binding affinity. Bold values indicate the lowest docking score for each target enzyme. Docking calculations were performed using optimized ligand conformations against the catalytic active-site regions of PGM/AlgC and HisIE enzymes. Meropenem was included as the reference antibacterial comparator. PGM/AlgC, Phosphoglucomutase/phosphomannomutase; HisIE, Bifunctional histidine biosynthesis enzyme.

In contrast, 5-Hydroxysophoranone showed the highest predicted affinity toward HisIE, with a docking score of −8.800 kcal/mol. HisIE is involved in bacterial histidine biosynthesis, a pathway associated with metabolic adaptation and survival under nutrient-limited or host-associated stress conditions. Thus, favorable binding of 5-Hydroxysophoranone within the HisIE binding groove may indicate potential disruption of amino-acid biosynthesis and adaptive bacterial metabolism. Isoforsythiaside showed comparatively weaker but still favorable binding energies against both receptors, suggesting that its antibacterial effect may be less dependent on strong direct target engagement and may require further optimization to improve potency.

Detailed residue-level interaction profiling further supported the biological relevance of the docking results. The selected compounds formed hydrogen bonds, hydrophobic contacts, electrostatic interactions, π-associated interactions, and van der Waals contacts with residues located within or near the predicted catalytic/binding regions (Supplementary Table 5, Figure 1, and Supplementary Figure 6). The presence of multiple stabilizing interactions within these functional pockets suggests that the observed docking scores were not only numerical predictions but reflected plausible receptor–ligand recognition patterns. In particular, the strong PGM/AlgC interaction profile of 10′-Desmethoxystreptonigrin was consistent with its stronger enzyme inhibition and antibacterial activity observed experimentally. Similarly, the favorable HisIE interaction profile of 5-Hydroxysophoranone supported its potential role as a complementary dual-target scaffold. These findings indicate that the selected natural compounds may suppress MDR respiratory pathogens through coordinated interference with metabolic enzymes involved in cell-envelope biosynthesis, stress adaptation, and virulence-associated persistence.

### Molecular dynamics and conformational stability analysis

**FIGURE 2:**
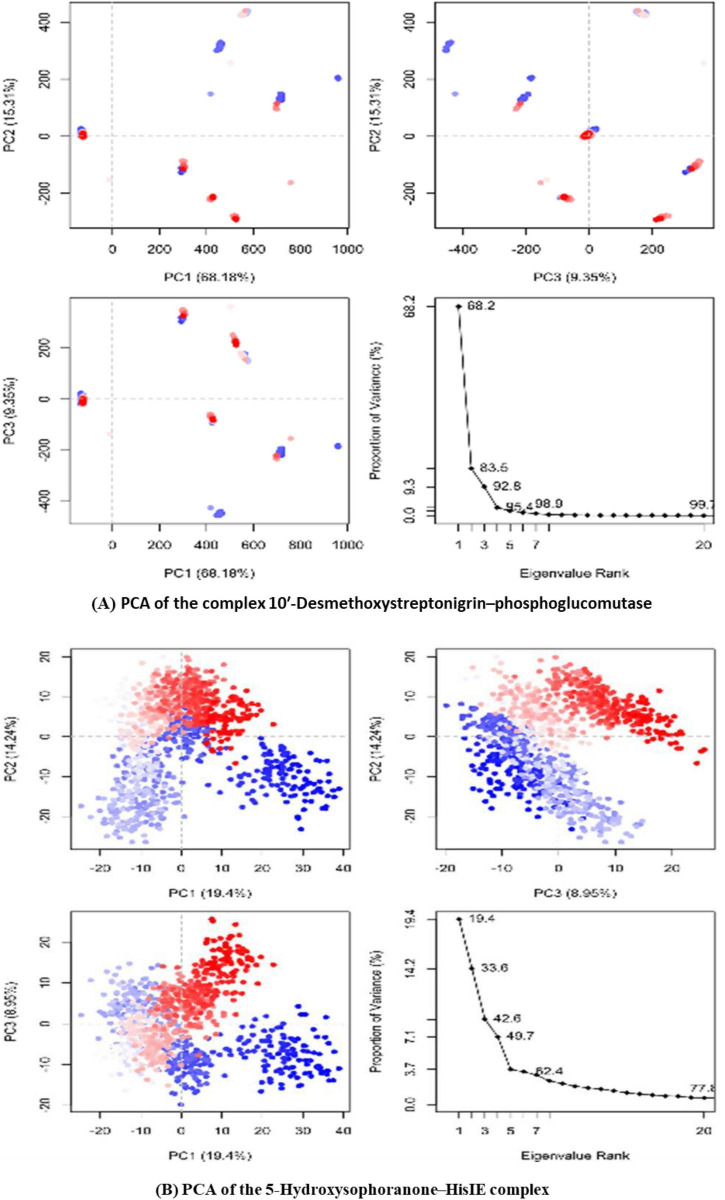
Principal component analysis (PCA) of lead compound–enzyme complexes during molecular dynamics simulations. **(A)** PCA projection plots and eigenvalue distribution of the 10′-Desmethoxystreptonigrin–PGM complex, showing conformational sampling and variance contribution of the principal components during the simulation trajectory. **(B)** PCA projection plots and eigenvalue distribution of the 5-Hydroxysophoranone–HisIE complex, illustrating ligand-associated conformational motions and variance distribution across the principal components. The PCA results demonstrated distinct but stable conformational sampling patterns for both enzyme–ligand complexes during molecular dynamics simulations.

Molecular dynamics simulations were performed for the two lead protein–ligand complexes, namely 10′-Desmethoxystreptonigrin–PGM/AlgC and 5-Hydroxysophoranone–HisIE, to evaluate the dynamic stability of the docking-predicted binding poses over 500 ns simulation trajectories (Figure 2 and Supplementary Figures 1, 2). RMSD analysis was used to assess global structural deviation and equilibration behavior of the protein–ligand complexes relative to their initial conformations. For the 10′-Desmethoxystreptonigrin–PGM/AlgC complex, the RMSD profile showed an initial adjustment phase during the early part of the simulation, followed by a more stable trajectory pattern, indicating that the complex reached conformational equilibration after initial structural relaxation. Importantly, no continuous RMSD increase or large-scale structural drift was observed during the later simulation period, suggesting that 10′-Desmethoxystreptonigrin remained stably accommodated within the PGM/AlgC catalytic pocket.

A similar trend was observed for the 5-Hydroxysophoranone–HisIE complex. The RMSD profile showed limited fluctuation after the initial equilibration phase, indicating that the HisIE receptor maintained a stable ligand-bound conformation during the production simulation. The absence of progressive RMSD elevation suggested that ligand binding did not induce major destabilization, unfolding-like deviation, or displacement from the HisIE binding groove. Together, the RMSD profiles supported the structural stability of both lead complexes and confirmed that the docking-derived binding poses were not transient or highly unstable during dynamic simulation.

RMSF analysis was performed to examine residue-level flexibility and to identify whether ligand binding induced localized instability in the receptor structures. In the 10′-Desmethoxystreptonigrin–PGM/AlgC complex, higher residue fluctuations were mainly localized in terminal and flexible loop regions, whereas residues located within or close to the catalytic pocket showed comparatively lower fluctuation. This pattern indicates that the PGM/AlgC binding pocket remained structurally stable during the simulation and that the observed flexibility was primarily associated with peripheral regions rather than the ligand-binding site. The reduced flexibility of pocket-associated residues further supports stable ligand accommodation and persistent protein–ligand interaction within the catalytic cleft.

For the 5-Hydroxysophoranone–HisIE complex, RMSF analysis also showed that the highest fluctuations were concentrated in flexible loop or terminal regions, while residues forming the predicted HisIE binding groove displayed comparatively restricted motion. This finding suggests that 5-Hydroxysophoranone binding did not destabilize the HisIE functional pocket and that the ligand-binding groove retained its structural integrity throughout the simulation. The comparatively stable RMSF behavior of binding-site residues was consistent with the protein–ligand contact analysis, which showed retention of selected hydrogen-bond, hydrophobic, and van der Waals interactions during the trajectory.

In addition to RMSD and RMSF analyses, radius of gyration and SASA profiles were used to assess structural compactness and solvent exposure. The radius of gyration profiles remained relatively stable for both lead complexes, indicating that the receptor structures maintained compact conformations without substantial expansion or collapse. SASA analysis showed no major progressive increase in solvent exposure, further supporting preservation of the folded receptor states during simulation. PCA projection plots also indicated restricted conformational sampling after equilibration, suggesting that both complexes remained within stable conformational spaces during the simulation period. Overall, the combined RMSD, RMSF, Rg, SASA, PCA, and protein–ligand contact analyses demonstrated that 10′-Desmethoxystreptonigrin–PGM/AlgC and 5-Hydroxysophoranone–HisIE formed dynamically stable complexes and retained favorable binding-pocket interactions throughout the MD simulations.

Detailed RMSD, RMSF, radius of gyration, SASA, and protein–ligand contact profiles for both simulated complexes are provided in Supplementary Figures 1, 2.

### Cytotoxicity assessment in respiratory epithelial cell lines

**FIGURE 3:**
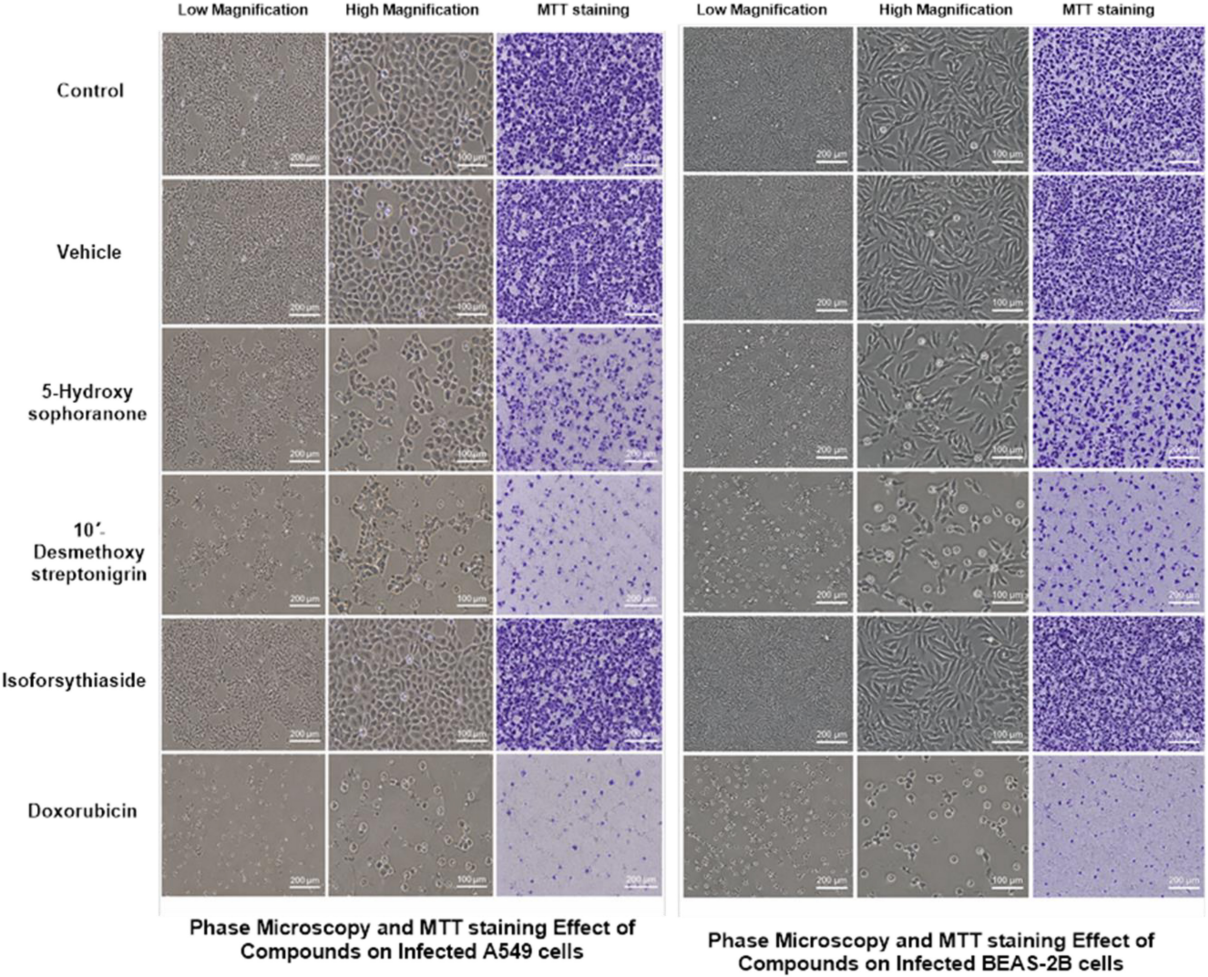
Phase-contrast microscopy and MTT staining of infected epithelial cells following treatment with selected compounds. **(A)** Representative phase-contrast microscopy and MTT staining images of infected A549 cells treated with vehicle, 5-Hydroxysophoranone, 10′-Desmethoxystreptonigrin, Isoforsythiaside, and Doxorubicin. **(B)** Representative phase-contrast microscopy and MTT staining images of infected BEAS-2B cells following treatment with vehicle, 5-Hydroxysophoranone, 10′-Desmethoxystreptonigrin, Isoforsythiaside, and Doxorubicin. Control and vehicle-treated cells maintained normal epithelial morphology and strong MTT staining, whereas compound-treated groups demonstrated variable effects on cell morphology and viability. Doxorubicin was used as a cytotoxic reference control. Scale bars: low magnification, 200 μm; high magnification, 100 μm; MTT staining, 200 μm.

The cytotoxic profiles of the selected compounds were evaluated in A549 and BEAS-2B epithelial cells following 48 h exposure (Table 2 and Figure 3). Vehicle-treated cells maintained viability comparable to untreated controls, confirming the absence of solvent-associated toxicity.

**TABLE 2:** Cytotoxic effects of selected compounds in A549 and BEAS-2B cells following 48 h exposure.

Compound	A549 cell viability (%)	BEAS-2B cell viability (%)	CC50 (μM)	Interpretation
Control	100.0 ± 2.8	100.0 ± 3.1	–	–
Vehicle	98.7 ± 2.4ns	97.9 ± 2.7ns	–	Non-toxic
5-Hydroxysophoranone	89.4 ± 3.2*	92.1 ± 2.8*	78.6 ± 4.1	Low cytotoxicity
10′-Desmethoxystreptonigrin	76.3 ± 4.5**	81.7 ± 3.9**	42.8 ± 3.6	Moderate cytotoxicity
Isoforsythiaside	94.8 ± 2.6ns	96.2 ± 2.3ns	96.4 ± 5.2	Low cytotoxicity
Doxorubicin	31.2 ± 2.9***	38.5 ± 3.3***	5.6 ± 0.7	Positive control

Values are presented as mean ± SD (*n* = 3). Statistical significance was determined relative to untreated control cells. ns, not significant;

*p < 0.05;

**p < 0.01;

***p < 0.001. CC50, 50% cytotoxic concentration; A549, Human alveolar epithelial cells; BEAS-2B, Human bronchial epithelial cells.

Isoforsythiaside exhibited the highest cytocompatibility, maintaining cell viabilities above 94% in both cell lines and demonstrating the highest CC50 value (96.4 ± 5.2 μM). 5-Hydroxysophoranone also showed relatively low cytotoxicity, with viabilities of 89.4 ± 3.2% in A549 cells and 92.1 ± 2.8% in BEAS-2B cells. In comparison, 10′-Desmethoxystreptonigrin produced moderate reductions in epithelial viability, although its cytotoxicity remained substantially lower than that of the positive control Doxorubicin.

Microscopic examination supported these findings, as control and vehicle-treated cells retained normal epithelial morphology and strong MTT staining, whereas Doxorubicin-treated cells exhibited pronounced cellular rounding, detachment, and reduced staining intensity (Figure 3).

### Antibacterial activity against multidrug-resistant respiratory pathogens

**FIGURE 4:**
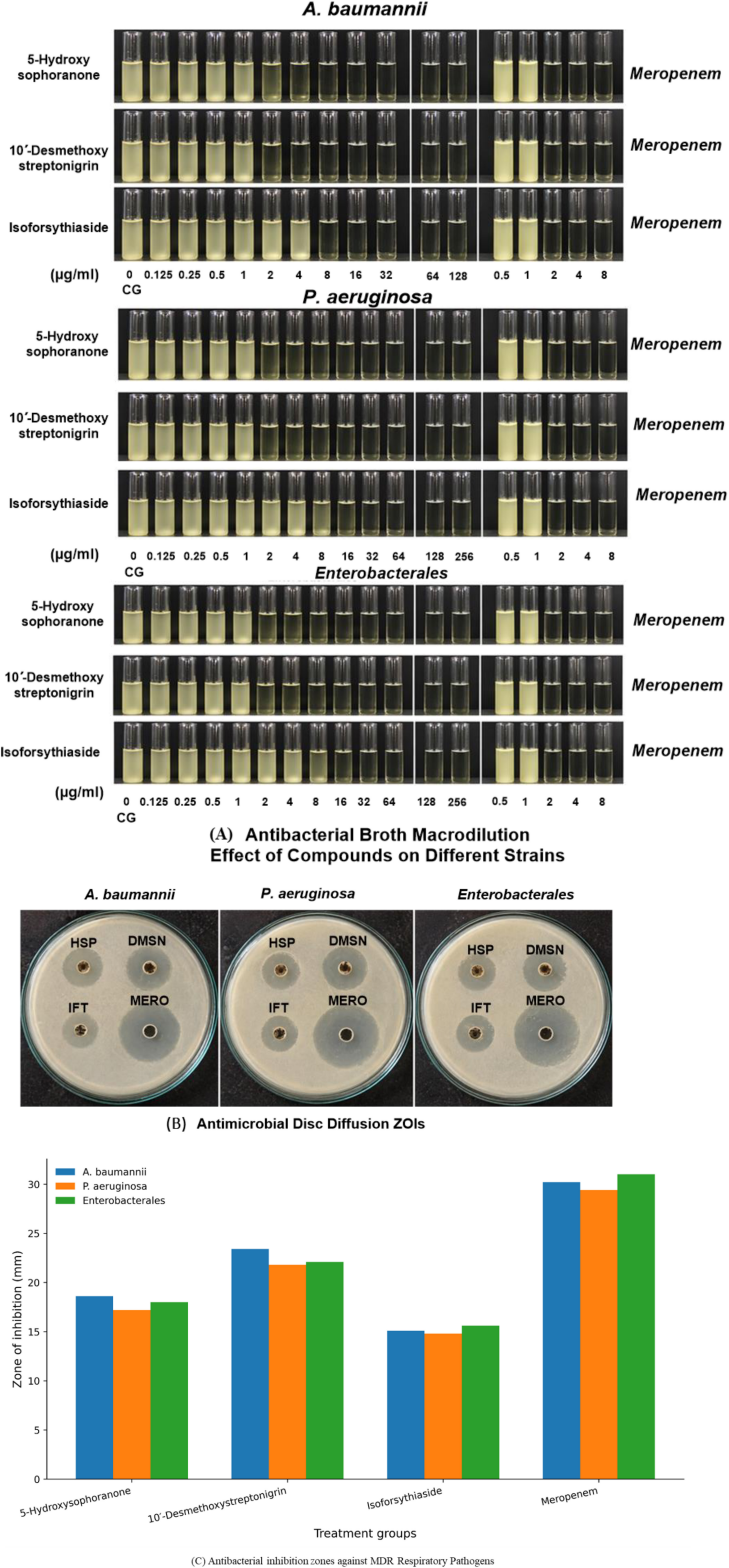
Antibacterial activity of selected compounds against multidrug-resistant respiratory pathogens. **(A)** Representative broth macrodilution assay showing concentration-dependent antibacterial activity of 5-Hydroxysophoranone, 10′-Desmethoxystreptonigrin, and Isoforsythiaside against *Acinetobacter baumannii*, *Pseudomonas aeruginosa*, and Enterobacterales isolates, with Meropenem used as the reference antibiotic. Reduced turbidity indicates inhibition of bacterial growth at increasing compound concentrations. **(B)** Representative agar disc diffusion assay demonstrating inhibition zones produced by 5-Hydroxysophoranone (HSP), 10′-Desmethoxystreptonigrin (DMSN), Isoforsythiaside (IFT), and Meropenem (MERO) against multidrug-resistant respiratory bacterial isolates. **(C)** Quantitative summary of inhibition-zone diameters produced by 5-Hydroxysophoranone, 10′-Desmethoxystreptonigrin, Isoforsythiaside, and Meropenem against *Acinetobacter baumannii*, *Pseudomonas aeruginosa*, and Enterobacterales isolates determined by disc diffusion assay. Values are presented as mean inhibition-zone diameter (mm) from three independent experiments. DMSO was used as the vehicle control. The tested compounds demonstrated measurable antibacterial activity with variable inhibitory effects across the examined bacterial strains.

The antibacterial activities of the selected compounds were evaluated against multidrug-resistant respiratory isolates using broth macrodilution and disc diffusion assays (Table 3 and Figure 4). Among the tested compounds, 10′-Desmethoxystreptonigrin demonstrated the strongest antibacterial activity, with MIC values ranging from 8 to 10 μg/mL and MBC values between 16 and 20 μg/mL across the tested pathogens. 5-Hydroxysophoranone showed intermediate antibacterial activity, whereas Isoforsythiaside required comparatively higher concentrations to achieve bacterial growth inhibition.

**TABLE 3:** Minimum inhibitory concentration (MIC), minimum bactericidal concentration (MBC), and selectivity index (SI) of selected compounds against multidrug-resistant respiratory pathogens.

Compound	*A. baumannii* MIC (μg/mL)	*P. aeruginosa* MIC (μg/mL)	Enterobacterales MIC (μg/mL)	MBC range (μg/mL)	Selectivity index (SI) range
5-Hydroxysophoranone	16.0 ± 1.2**	20.0 ± 1.4**	18.0 ± 1.1**	32–40	3.9–4.9
10′-Desmethoxystreptonigrin	8.0 ± 0.6***	10.0 ± 0.8***	9.0 ± 0.7***	16–20	4.3–5.4
Isoforsythiaside	40.8 ± 2.1**	40.8 ± 2.4**	48.0 ± 2.9**	64–80	2.0–2.4
Meropenem	2.0 ± 0.2***	2.0 ± 0.2***	2.0 ± 0.2***	4–8	–

Values are presented as mean ± SD (*n* = 3). Statistical significance was determined relative to untreated bacterial controls.

**p < 0.01;

***p < 0.001. Selectivity index (SI) was calculated using the ratio of CC50 to MIC values. MIC, Minimum inhibitory concentration; MBC, Minimum bactericidal concentration; *A. baumannii*, *Acinetobacter baumannii*; *P. aeruginosa*, *Pseudomonas aeruginosa*; SI, Selectivity index.

Disc diffusion analysis demonstrated a similar activity trend, with 10′-Desmethoxystreptonigrin producing the largest inhibition zones among the natural compounds tested (Supplementary Table 2 and Figures 4B,C). Meropenem consistently exhibited the highest antibacterial efficacy, while DMSO controls showed no measurable inhibitory effect.

Collectively, these findings confirmed measurable extracellular antibacterial activity of the selected compounds against multidrug-resistant respiratory pathogens.

### Cell-associated antibacterial activity in infected epithelial cells

The cell-associated antibacterial efficacy of the compounds was examined using infected A549 epithelial cells (Table 4). Treatment with 10′-Desmethoxystreptonigrin resulted in the greatest reduction in bacterial burden, decreasing cell-associated bacterial counts by 3.51 log10 CFU/mL relative to infected untreated controls. 5-Hydroxysophoranone and Isoforsythiaside also reduced cell-associated bacterial survival.

**TABLE 4:** Cell-associated antibacterial activity of selected compounds in infected A549 cells after 24 h treatment.

Treatment group	Bacterial burden (log10 CFU/mL)	Reduction vs. infected control (log10 CFU/mL)
Infected control	7.82 ± 0.18	–
5-Hydroxysophoranone	5.14 ± 0.21**	2.68
10′-Desmethoxystreptonigrin	4.31 ± 0.17***	3.51
Isoforsythiaside	5.76 ± 0.24**	2.06
Meropenem	3.22 ± 0.14***	4.60

Values are presented as mean ± SD (*n* = 3). Statistical significance was determined relative to infected untreated control cells.

**p < 0.01;

***p < 0.001. CFU, Colony-forming units; A549, Human alveolar epithelial cells.

Meropenem produced the strongest overall cell-associated antibacterial effect, with a 4.60 log10 CFU/mL reduction. These observations indicated that the selected compounds retained antibacterial activity within infected epithelial environments.

### Inhibition of PGM/AlgC and HisIE enzymatic activity

Enzyme inhibition assays demonstrated concentration-dependent inhibitory activity of the tested compounds against both PGM/AlgC and HisIE enzymes (Table 5). 10′-Desmethoxystreptonigrin showed the strongest inhibitory effect among the natural compounds, with IC50 values of 3.21 ± 0.19 μM for PGM/AlgC and 3.88 ± 0.22 μM for HisIE. In contrast, Isoforsythiaside displayed comparatively weaker inhibition profiles.

**TABLE 5:** Inhibitory effects of selected compounds against PGM/AlgC and HisIE enzymes.

Treatment group	PGM/AlgC IC50 (μM)	HisIE IC50 (μM)	Enzyme inhibition at 25 μM (%)
Control	–	–	0.0 ± 0.0
Meropenem	2.11 ± 0.14***	2.38 ± 0.16***	91.6 ± 2.1***
5-Hydroxysophoranone	7.42 ± 0.38**	8.11 ± 0.41**	73.8 ± 2.6**
10′-Desmethoxystreptonigrin	3.21 ± 0.19***	3.88 ± 0.22***	86.4 ± 2.2***
Isoforsythiaside	11.36 ± 0.64*	12.91 ± 0.72*	61.7 ± 2.9*

Values are presented as mean ± SD (*n* = 3). Statistical significance was determined relative to untreated control samples.

*p < 0.05;

**p < 0.01;

***p < 0.001. IC50, Half-maximal inhibitory concentration; PGM/AlgC, Phosphoglucomutase/phosphomannomutase; HisIE, Bifunctional histidine biosynthesis enzyme.

At 25 μM, 10′-Desmethoxystreptonigrin achieved 86.4 ± 2.2% enzyme inhibition, approaching the activity of Meropenem (91.6 ± 2.1%). These findings were consistent with the molecular docking and molecular dynamics analyses, supporting stable interactions between the lead compounds and both metabolic targets.

### Modulation of metabolic and virulence-associated markers

**FIGURE 5:**
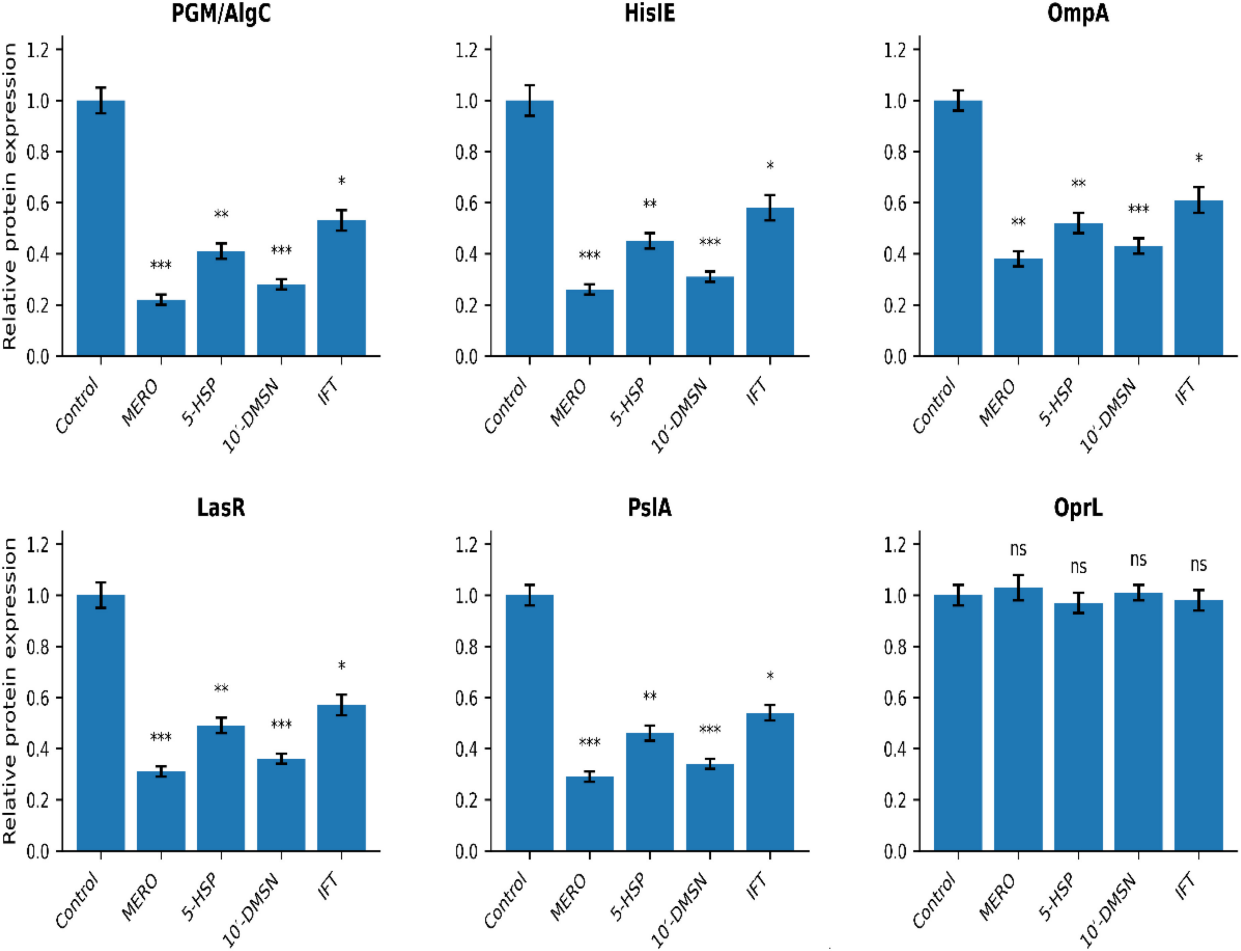
Densitometric quantification of Western blot band intensities for PGM/AlgC, HisIE, OmpA, LasR, PslA, and OprL following compound treatment. Protein levels were normalized to GroEL and expressed relative to the untreated control. Data are presented as mean ± SD from three independent experiments. ns, not significant; *p < 0.05; **p < 0.01; ***p < 0.001 versus untreated control. MERO, Meropenem; 5-HSP, 5-Hydroxysophoranone; 10′-DMSN, 10′-Desmethoxystreptonigrin; IFT, Isoforsythiaside.

Western blot analysis demonstrated substantial reductions in the expression of multiple metabolic and virulence-associated proteins following compound treatment (Table 6, Figure 5 and Supplementary Figure 3). Densitometric quantification of three independent Western blot experiments confirmed that the strongest suppression occurred in the 10′-Desmethoxystreptonigrin-treated group, particularly for PGM/AlgC, HisIE, LasR, PslA, and OmpA. Relative to the untreated control, 10′-Desmethoxystreptonigrin reduced PGM/AlgC, HisIE, OmpA, LasR, and PslA expression to 0.28 ± 0.02, 0.31 ± 0.02, 0.43 ± 0.03, 0.36 ± 0.02, and 0.34 ± 0.02, respectively. Moderate reductions were observed following treatment with 5-Hydroxysophoranone, whereas Isoforsythiaside produced comparatively weaker effects. GroEL expression remained largely unchanged across treatment groups, supporting comparable protein loading. Representative triplicate Western blot images are provided in Supplementary Figure 3 to demonstrate reproducibility.

**TABLE 6:** Densitometric quantification of Western blot protein-expression levels following compound treatment.

Protein	Control	Meropenem	5-Hydroxysophoranone	10′-Desmethoxystreptonigrin	Isoforsythiaside
oprL	1.00 ± 0.04ns	1.03 ± 0.05ns	0.97 ± 0.04ns	1.01 ± 0.03ns	0.98 ± 0.04ns
pgm/algC	1.00 ± 0.05	0.22 ± 0.02***	0.41 ± 0.03**	0.28 ± 0.02***	0.53 ± 0.04*
HisIE	1.00 ± 0.06	0.26 ± 0.02***	0.45 ± 0.03**	0.31 ± 0.02***	0.58 ± 0.05*
ompA	1.00 ± 0.04	0.38 ± 0.03**	0.52 ± 0.04**	0.43 ± 0.03***	0.61 ± 0.05*
lasR	1.00 ± 0.05	0.31 ± 0.02***	0.49 ± 0.03**	0.36 ± 0.02***	0.57 ± 0.04*
pslA	1.00 ± 0.04	0.29 ± 0.02***	0.46 ± 0.03**	0.34 ± 0.02***	0.54 ± 0.03*
GroEL	1.00 ± 0.04ns	0.99 ± 0.03ns	1.01 ± 0.04ns	0.98 ± 0.03ns	0.04ns

Values are presented as mean ± SD (*n* = 3) and normalized relative to untreated control samples. Statistical significance was determined relative to untreated control groups. ns, not significant;

*p < 0.05;

**p < 0.01;

***p < 0.001. pgm/algC, Phosphoglucomutase/Phosphomannomutase; ompA, Outer membrane protein A; lasR, Quorum-sensing transcriptional regulator; pslA, Polysaccharide biosynthesis protein; oprL, Outer membrane lipoprotein; GroEL, Housekeeping chaperone protein.

qPCR analysis showed a parallel transcriptional response (Supplementary Table 4 and Supplementary Figure 4). Significant downregulation of genes associated with carbohydrate metabolism, quorum sensing, biofilm formation, oxidative stress adaptation, and bacterial persistence was observed following treatment with the tested compounds. qPCR analysis showed a parallel transcriptional response (Supplementary Table 4 and Supplementary Figure 4). Significant downregulation of genes associated with carbohydrate metabolism, quorum sensing, biofilm formation, oxidative stress adaptation, and bacterial persistence was observed following treatment with the tested compounds. The strongest transcriptional suppression was again detected in the 10′-Desmethoxystreptonigrin group. The combined Western blot and qPCR results are presented in Supplementary Figure 5.

### Integrated activity profiling and biological interpretation

**FIGURE 6:**
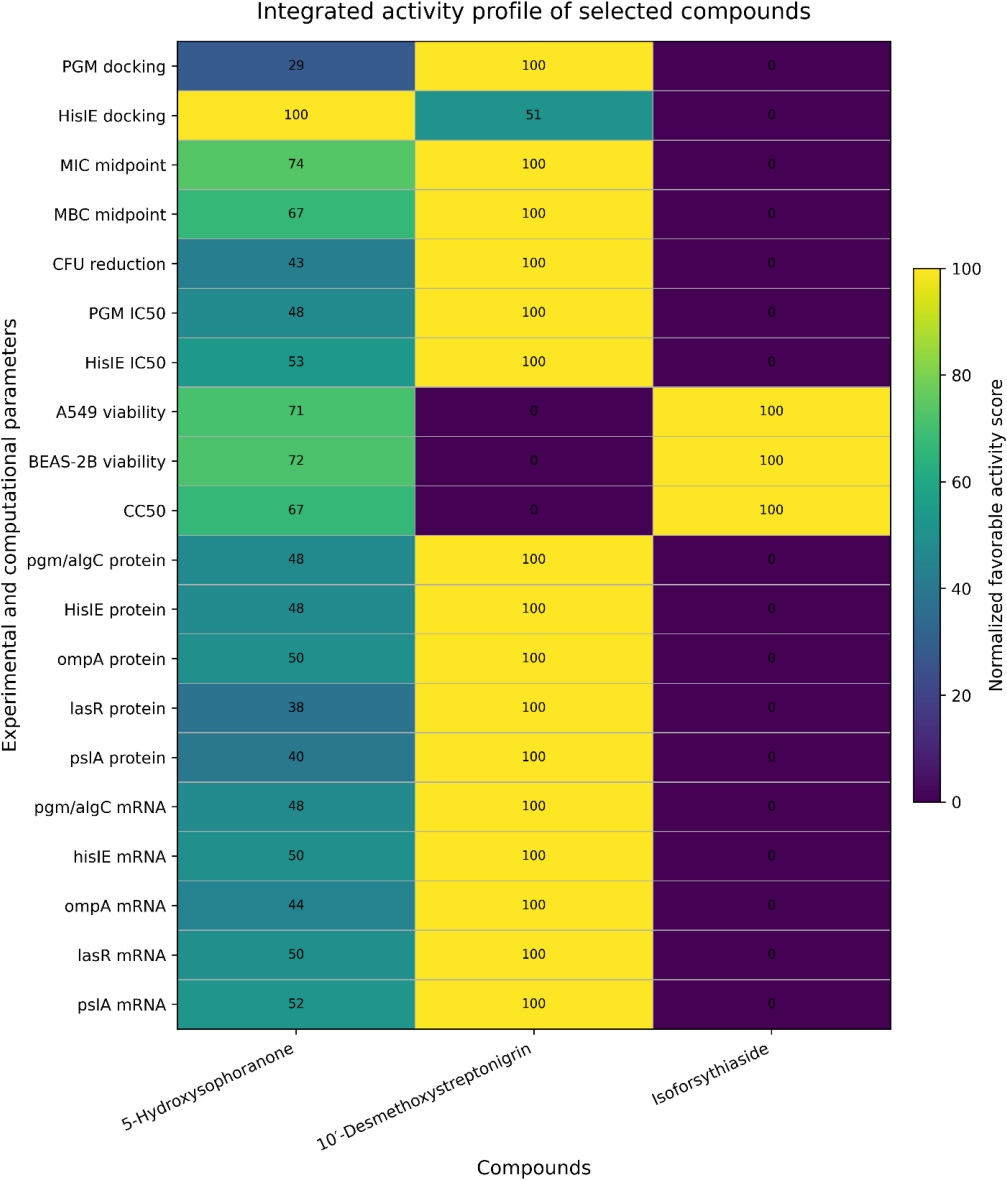
Integrated heatmap analysis of computational, antibacterial, enzymatic, and molecular activity profiles of selected compounds. Heatmap visualization summarizing the relative performance of 5-Hydroxysophoranone, 10′-Desmethoxystreptonigrin, and Isoforsythiaside across multiple computational and experimental parameters, including docking affinity, antibacterial activity, enzyme inhibition, cytocompatibility, cell-associated bacterial reduction, and suppression of metabolic and virulence-associated markers. Color intensity represents normalized favorable activity scores, with higher values indicating stronger overall activity or biological performance for the corresponding parameter. The integrated analysis highlights distinct activity patterns among the tested compounds across antibacterial, mechanistic, and cytotoxicity-related datasets.

Integrated heatmap analysis summarized the relative computational, antibacterial, enzymatic, molecular, and cytocompatibility profiles of the tested compounds (Figure 6 and Table 7). The integrated computational and experimental profiles are further summarized in Supplementary Table 7. This integrated analysis was included to determine whether the computationally predicted target interactions were supported by downstream biological responses. Among the selected compounds, 10m-Desmethoxystreptonigrin consistently showed the strongest overall activity profile, including the most favorable PGM/AlgC docking score, stable ligand accommodation during MD simulation, the lowest MIC/MBC values, the greatest cell-associated bacterial reduction, the strongest enzyme inhibition, and the greatest suppression of metabolic and virulence-associated markers. This convergence of computational and experimental evidence suggests that 10s-Desmethoxystreptonigrin may act as the most potent antibacterial lead compound in this study.

**TABLE 7:** Comparative translational profile of lead compounds against multidrug-resistant respiratory pathogens.

Parameter	5-Hydroxysophoranone	10′-Desmethoxystreptonigrin	Isoforsythiaside
Docking affinity	Strong	Very strong	Moderate
Antibacterial activity	Moderate-to-strong	Strong	Moderate
cell-associated antibacterial activity	Moderate	Strong	Moderate
PGM/AlgC inhibition	Moderate	Strong	Weak-to-moderate
HisIE inhibition	Moderate	Strong	Weak-to-moderate
Virulence-gene suppression	Moderate	Strong	Moderate
Cytocompatibility	High	Moderate	Very high
Overall therapeutic profile	Balanced activity and safety	Highest antibacterial potency	Highest cytocompatibility

The biological significance of these findings is that the computational results were consistent with functional antibacterial outcomes. The strong predicted interaction of 10′-Desmethoxystreptonigrin with PGM/AlgC was supported by reduced PGM/AlgC protein expression, lower pgm/algC transcription, stronger enzyme inhibition, and reduced bacterial burden. Because PGM/AlgC is associated with carbohydrate metabolism, surface polysaccharide production, and cell-envelope-related virulence, inhibition of this pathway may contribute to impaired bacterial adaptation and reduced persistence. Similarly, HisIE-associated interactions were biologically relevant because reduced HisIE activity and hisIE expression may affect histidine biosynthesis and stress-associated metabolic survival.

**FIGURE 7:**
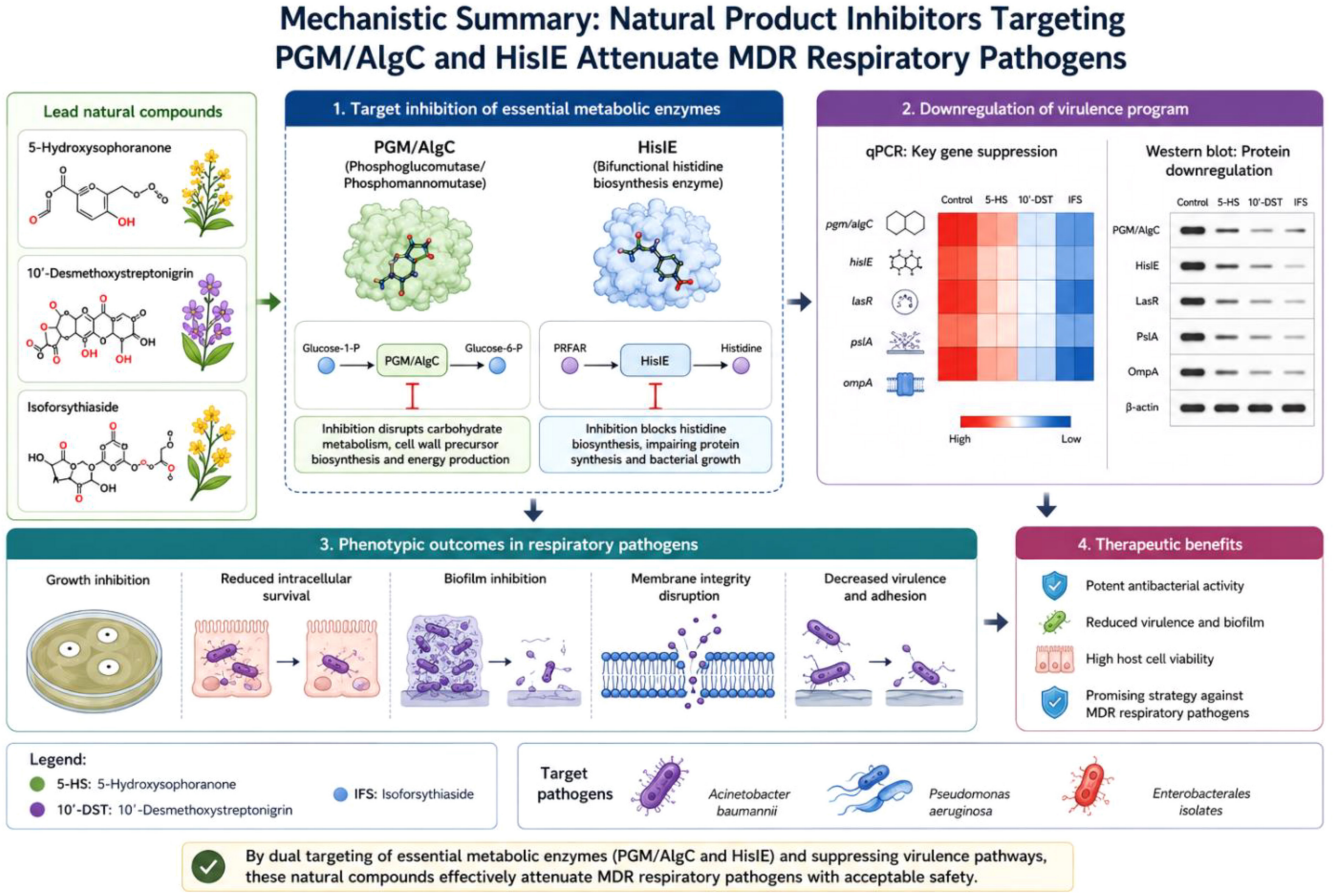
Proposed mechanistic model of dual-target inhibition mediated by lead natural compounds against multidrug-resistant respiratory pathogens. Schematic representation of the proposed antibacterial mechanism of 5-Hydroxysophoranone, 10′-Desmethoxystreptonigrin, and Isoforsythiaside against multidrug-resistant respiratory pathogens. The compounds demonstrated interactions with PGM/AlgC and HisIE enzymes, resulting in suppression of metabolic and virulence-associated pathways, including quorum sensing, biofilm-associated signaling, and outer membrane integrity. Molecular and phenotypic analyses indicated reduced bacterial survival, decreased cell-associated persistence, attenuation of virulence-associated markers, and preservation of host-cell viability. The integrated findings support the potential of dual-target natural product inhibitors as candidate therapeutic agents against multidrug-resistant respiratory infections.

Although Isoforsythiaside showed weaker docking and antibacterial activity, it demonstrated the highest epithelial-cell compatibility, indicating that it may be more useful as a safer scaffold requiring potency optimization. 5-Hydroxysophoranone showed a more balanced activity–safety profile, combining favorable HisIE docking, moderate antibacterial activity, and acceptable cytocompatibility. Overall, the combined computational and experimental findings support a proposed dual-target inhibitory mechanism involving suppression of PGM/AlgC- and HisIE-associated pathways, leading to impaired bacterial metabolism, reduced virulence-associated signaling, decreased cell-associated bacterial persistence, and attenuation of MDR respiratory pathogens (Figure 7).

## Discussion

The increasing prevalence of multidrug-resistant (MDR) Gram-negative respiratory pathogens has intensified the need for alternative antibacterial strategies that extend beyond conventional bactericidal targets (Murray et al., 2022; Ventola, 2015). In the present study, an integrated computational and experimental framework was used to evaluate selected natural compounds against two metabolically relevant bacterial enzymes, PGM/AlgC and HisIE, which are associated with carbohydrate metabolism, stress adaptation, and virulence regulation. The findings demonstrated that the tested compounds, particularly 10′-Desmethoxystreptonigrin and 5-Hydroxysophoranone, exhibited measurable antibacterial activity together with inhibitory effects on metabolic and virulence-associated pathways in representative MDR respiratory pathogens.

Among the evaluated compounds, 10′-Desmethoxystreptonigrin consistently demonstrated the strongest computational and experimental activity profile. This compound exhibited the most favorable docking affinity toward PGM/AlgC, stable ligand accommodation during molecular dynamics simulations, comparatively low MIC values, and substantial enzyme inhibition. The observed reduction in cell-associated bacterial burden further suggested that the compound retained antibacterial activity within infected epithelial environments. Previous studies have shown that disruption of carbohydrate-associated metabolic pathways may impair membrane biosynthesis, biofilm maturation, and bacterial persistence in *P. aeruginosa* and related Gram-negative organisms (Akter et al., 2025; Colvin et al., 2011). The present findings are consistent with these observations and support the concept that interference with central metabolic pathways may contribute to antibacterial activity against MDR respiratory pathogens.

The PGM/AlgC pathway represents an attractive antibacterial target because of its involvement in lipopolysaccharide synthesis, alginate production, and exopolysaccharide-mediated biofilm formation (Liang et al., 2022). In Gram-negative respiratory pathogens, these processes are closely associated with chronic colonization, immune evasion, and reduced antibiotic penetration (Singh et al., 2023). The observed downregulation of *pgm/algC*, *pslA*, *ompA*, and *lasR* at both protein and transcriptional levels suggests that the tested compounds may interfere with pathways linked to biofilm-associated persistence and quorum sensing. Although the current study did not directly quantify biofilm biomass or quorum-sensing metabolites, the molecular-expression profiles support possible modulation of virulence-associated signaling networks.

The bifunctional HisIE enzyme was selected as a complementary metabolic target because histidine biosynthesis remains essential for bacterial adaptation under nutrient-limited and stress-associated conditions (Zlitni et al., 2013). Histidine biosynthetic pathways have previously been linked to oxidative stress tolerance and survival during host infection (Imber et al., 2018). In the present study, compounds showing stronger HisIE interactions also demonstrated reductions in stress-associated markers such as *katG* and *dnaK*. These findings may indicate broader metabolic perturbation following target inhibition, although further mechanistic validation will be required to establish direct pathway-specific effects.

Natural products continue to represent an important source of structurally diverse antimicrobial scaffolds (Nasim et al., 2022). The natural compounds evaluated in this study contain multiple hydroxyl, quinone, and glycosidic functional groups capable of establishing hydrogen-bonding and electrostatic interactions within catalytic enzyme pockets. Previous investigations have reported antibacterial and enzyme-modulatory activities for related flavonoid and quinone-containing compounds against Gram-negative pathogens (Shamsudin et al., 2022; Thebti et al., 2023). Consistent with these reports, the present study demonstrated that structurally distinct natural compounds may exhibit differential activity profiles, with 10′-Desmethoxystreptonigrin showing stronger antibacterial potency and Isoforsythiaside demonstrating comparatively higher cytocompatibility.

The integrated use of molecular docking, molecular dynamics simulations, MM/GBSA analysis, enzyme inhibition assays, and molecular-expression studies strengthened the mechanistic interpretation of the findings. In particular, the MD simulations provided dynamic support for the docking results. RMSD profiles indicated that the lead protein–ligand complexes reached stable conformational states after initial equilibration, suggesting that the docked poses were not transient or highly unstable. RMSF analysis showed that the highest residue fluctuations were mainly localized in terminal or loop regions, whereas the binding-pocket regions remained comparatively stable, supporting sustained ligand accommodation in functionally relevant sites. The radius of gyration profiles suggested that the protein structures retained compact conformations during simulation, while SASA analysis showed no major progressive increase in solvent exposure, indicating that ligand binding did not promote unfolding-like structural expansion (Hollingsworth and Dror, 2018; Mulcahy et al., 2014; Lionta et al., 2014).

These MD-derived observations are important because docking provides only a static binding-pose prediction, whereas MD simulation evaluates whether the predicted ligand–target interactions remain stable under dynamic conditions. The retention of selected hydrogen-bond, hydrophobic, and van der Waals contacts during MD simulation further supports the stability of the 10′-Desmethoxystreptonigrin–PGM/AlgC and 5-Hydroxysophoranone–HisIE complexes. Therefore, the combined docking and MD results provide stronger evidence that the selected natural compounds can remain accommodated within the catalytic/binding pockets of PGM/AlgC and HisIE. Nevertheless, these computational findings should be interpreted as supportive mechanistic evidence and not as a substitute for biochemical target-validation assays, which were therefore included in the experimental part of this study.

Recent computational drug-discovery studies have increasingly combined molecular docking, molecular dynamics simulation, and binding-energy analysis to improve the reliability of ligand prioritization. For example, Sahin and Ali used an AI-driven ligand-optimization workflow and emphasized that docking provides a static approximation of ligand binding, whereas MD simulation is required to assess dynamic stability and persistence of protein–ligand interactions under time-dependent conditions. Similarly, Saleem et al. reported an integrated *in silico* workflow for Moringa oleifera phytochemicals targeting TCF7L2 and interpreted RMSD, ligand RMSD, binding-pocket RMSD, Rg, SASA, hydrogen-bond stability, and PCA to support complex convergence and structural stability during MD simulation. These studies are consistent with the present work in showing that docking scores alone are insufficient and should be supported by dynamic stability parameters such as RMSD, RMSF, Rg, SASA, PCA, and protein–ligand contact persistence (Ali et al., 2026; Javaid et al., 2026; Sahin and Ali, 2026; Saleem et al., 2026).

The present findings also align with recent natural-product screening studies in which phytochemical libraries were first evaluated computationally and then prioritized based on binding behavior, ADME-related properties, interaction stability, and molecular-dynamics performance. For instance, a recent Scientific Reports study screened a phytochemical library against FGFR3 and used docking validation, MD simulation, MM/GBSA analysis, and residue-level interaction interpretation to prioritize a natural inhibitor candidate. In antibacterial research, Ali et al. applied an integrated ethnobotanical and *in silico* approach to identify plant-derived compounds targeting the virulence-associated fmhA/FemA system in Staphylococcus warneri, combining phytochemical identification, PubChem-based ligand retrieval, docking interaction analysis, 3D visualization, MD simulation, RMSD/RMSF interpretation, Rg, hydrogen-bond, and SASA analyses. These reports support the value of integrating docking with residue-level visualization and MD-based stability assessment for natural-product lead identification (Ali et al., 2026; Javaid et al., 2026; Sahin and Ali, 2026; Saleem et al., 2026).

However, the present study differs from these previous reports in several important aspects. First, instead of focusing on a single receptor, the present work examined a dual-target antibacterial strategy involving PGM/AlgC and HisIE, two bacterial metabolic enzymes associated with carbohydrate metabolism, cell-envelope biosynthesis, histidine biosynthesis, stress adaptation, and virulence-associated persistence. Second, the computational predictions were not interpreted as standalone evidence; they were connected to antibacterial susceptibility testing, cell-associated bacterial reduction, direct enzyme inhibition, Western blotting, and qPCR-based expression analysis. This is important because several computational studies primarily prioritize compounds based on docking and MD outputs, whereas the present study provides experimental support showing that the strongest computationally predicted compound, 10′-Desmethoxystreptonigrin, also produced the strongest antibacterial, enzyme-inhibitory, and molecular-suppression effects. Third, the study highlights a biologically meaningful activity–toxicity balance: 10′-Desmethoxystreptonigrin showed the greatest antibacterial potency but moderate cytotoxicity, whereas Isoforsythiaside showed higher epithelial-cell compatibility but weaker target inhibition. This comparison emphasizes that computational binding strength must be interpreted together with biological activity and host-cell tolerance.

Therefore, the novelty of the present work lies not simply in applying molecular docking and MD simulation, but in using an integrated computational–experimental framework to connect dual-target binding predictions with functional antibacterial outcomes and modulation of virulence-associated pathways in MDR respiratory pathogens. The findings extend previous natural-product computational screening studies by demonstrating that simultaneous interference with PGM/AlgC- and HisIE-associated pathways may provide a complementary strategy for attenuating bacterial metabolism, persistence, and virulence-associated signaling. Nevertheless, consistent with limitations noted in recent computational drug-discovery studies, further validation using broader clinical isolate panels, direct target-binding assays, biofilm and quorum-sensing assays, pharmacokinetic evaluation, and *in vivo* respiratory infection models will be required before therapeutic translation can be established.

From a microbiological perspective, the cell-associated infection experiments are particularly relevant because respiratory pathogens such as *P. aeruginosa* and *A. baumannii* may persist within epithelial environments and biofilm-associated niches where antibiotic penetration is reduced (Mulcahy et al., 2014). The observed reductions in cell-associated bacterial burden suggest that the selected compounds retain activity under host-associated conditions. However, the antibacterial effects observed *in vitro* remain moderate when compared with standard antibiotics such as Meropenem, indicating that additional optimization would be necessary before translational application could be considered.

The cytotoxicity findings also provide important context regarding the potential therapeutic window of the tested compounds. Isoforsythiaside demonstrated the highest epithelial-cell compatibility, whereas 10′-Desmethoxystreptonigrin exhibited stronger antibacterial activity but comparatively lower host-cell tolerance. This activity–toxicity balance is frequently encountered during antimicrobial lead optimization and highlights the importance of evaluating both antibacterial potency and host-cell compatibility during early drug-discovery stages (Sahin and Ali, 2026).

### Mechanistic significance of dual-target inhibition

The computational findings support the proposed dual-target mechanism by showing that the selected compounds interact with biologically relevant binding regions of both PGM/AlgC and HisIE. This is important because PGM/AlgC is associated with carbohydrate metabolism, cell-envelope biosynthesis, alginate production, and virulence-related persistence, whereas HisIE is linked to histidine biosynthesis and bacterial metabolic adaptation. The strong docking affinity and MD stability of 10′-Desmethoxystreptonigrin, particularly against PGM/AlgC, were consistent with its stronger antibacterial activity, enzyme inhibition, cell-associated bacterial reduction, and suppression of metabolic and virulence-associated markers. These findings suggest that the compound may weaken MDR respiratory pathogens by interfering with both metabolic function and virulence-associated survival pathways. Therefore, the dual-target strategy may provide a useful framework for developing natural-product-derived antibacterial leads, although further optimization is required to improve potency, selectivity, and safety.

### Study limitations

Several limitations should be acknowledged. First, the study was restricted to *in vitro* and computational analyses, and no *in vivo* infection models were included to evaluate pharmacokinetics, toxicity, or therapeutic efficacy under physiological conditions. Second, only a limited number of representative MDR respiratory isolates were examined, and broader strain diversity would strengthen microbiological interpretation. Third, although the molecular-expression data suggested modulation of virulence-associated pathways, direct evaluation of biofilm formation, quorum-sensing activity, and membrane permeability was not performed. In addition, the current work focused primarily on target-oriented inhibition and did not investigate possible synergistic interactions with clinically used antibiotics.

Future studies should therefore include *in vivo* respiratory infection models, expanded antimicrobial susceptibility profiling, direct biofilm quantification assays, and structural optimization of the lead compounds to improve potency and selectivity.

## Conclusion

The present study demonstrated that selected natural compounds possess measurable dual-target inhibitory activity against the bacterial metabolic enzymes PGM/AlgC and HisIE in representative MDR respiratory pathogens. Integrated computational, enzymatic, microbiological, and molecular analyses indicated that 10′-Desmethoxystreptonigrin exhibited the strongest antibacterial and enzyme-inhibitory activity, whereas Isoforsythiaside showed comparatively higher cytocompatibility. The suppression of pgm/algC, hisIE, lasR, pslA, ompA, and related markers suggests that interference with bacterial metabolic adaptation and virulence-associated pathways may contribute to the observed antibacterial effects.

The main message of this study is that natural-product-derived compounds may serve as useful starting scaffolds for developing antibacterial strategies that target both bacterial metabolism and virulence regulation. Rather than relying only on direct growth inhibition, this approach may help attenuate bacterial persistence, cell-associated survival, and adaptive pathogenic responses. However, the present findings should be interpreted as early-stage evidence. Further studies using broader clinical isolate panels, direct biofilm and quorum-sensing assays, *in vivo* respiratory infection models, pharmacokinetic assessment, and structural optimization are required before translational or therapeutic application can be established.

## Data Availability

The original contributions presented in this study are included in the article/Supplementary material, further inquiries can be directed to the corresponding author.
