# Supplementary material for: Integrated computational and experimental evaluation of natural products targeting PGM/AlgC and HisIE in multidrug-resistant respiratory pathogens
Source: Front Microbiol. 2026 Aug 3;17:1891583. doi: 10.3389/fmicb.2026.1891583 (PMC13478258; doi:10.3389/fmicb.2026.1891583)
Supplement: Supplementary file 1 [file Data_Sheet_1.docx]

**SUPPLEMENTARY FILE**

**Table S1**: **MM/GBSA binding free-energy components of lead compound–enzyme complexes during molecular dynamics simulations**

| **Energy component** | **PGM–10′-Desmethoxystreptonigrin (0 ns)** | **PGM–10′-Desmethoxystreptonigrin (500 ns)** | **HisIE–5-Hydroxysophoranone (0 ns)** | **HisIE–5-Hydroxysophoranone (500 ns)** |
| --- | --- | --- | --- | --- |
| **ΔGbind (kcal/mol)** | −280.56 | −209.81 | −58.57 | −24.07 |
| **Coulomb (kcal/mol)** | −87.90 | −80.00 | −15.22 | −20.93 |
| **Covalent (kcal/mol)** | 15.80 | 7.82 | 2.39 | 3.74 |
| **H-bond (kcal/mol)** | −5.58 | −6.94 | −1.08 | −0.93 |
| **Lipophilic (kcal/mol)** | −111.72 | −77.01 | −33.61 | −6.00 |
| **SolvGB (kcal/mol)** | 61.21 | 58.50 | 35.77 | 22.10 |
| **Ligand efficiency** | −2.08 | −1.55 | −1.67 | −0.69 |

- MM/GBSA calculations were performed using representative frames extracted from molecular dynamics trajectories. More negative ΔGbind values indicate stronger predicted binding affinity.
- MM/GBSA: Molecular mechanics/generalized Born surface area; ΔGbind: Binding free energy; SolvGB: Generalized Born solvation energy; PGM: Phosphoglucomutase.

**Table S2**: **Disc diffusion antibacterial activity of selected compounds against multidrug-resistant respiratory pathogens**

| **Compound** | **A. baumannii (mm)** | **P. aeruginosa (mm)** | **Enterobacterales (mm)** |
| --- | --- | --- | --- |
| 5-Hydroxysophoranone | 18.6 ± 0.9** | 17.2 ± 1.0** | 18.0 ± 0.8** |
| 10′-Desmethoxystreptonigrin | 23.4 ± 1.1*** | 21.8 ± 1.2*** | 22.1 ± 0.9*** |
| Isoforsythiaside | 15.1 ± 0.7* | 14.8 ± 0.8* | 15.6 ± 0.9* |
| Meropenem | 30.2 ± 1.3*** | 29.4 ± 1.1*** | 31.0 ± 1.4*** |

- Values are presented as mean inhibition-zone diameter ± SD (n = 3).
- Statistical significance was determined relative to untreated bacterial controls. *p < 0.05; **p < 0.01; ***p < 0.001.
- A. baumannii: Acinetobacter baumannii; P. aeruginosa: Pseudomonas aeruginosa.

**Table S3**: **Primer sequences used for quantitative real-time PCR analysis**

| **Gene** | **Forward primer (5′–3′)** | **Reverse primer (5′–3′)** | **Product size (bp)** |
| --- | --- | --- | --- |
| pgm/algC | ATGACCGACCTGCTGATCAA | TGGTAGGTGATGTCGTTGGC | 152 |
| hisIE | CGCTACGACATCGACAACTT | GGTAGCGATGGTGATGTCGT | 148 |
| ompA | TCGGTGCTGATGTTGCTGAT | AGCAGTTGGTGGTGATGGTT | 145 |
| lasR | GACCTGCTGCTCAAGGACTT | TTGATCGACGGTGATCTGGT | 150 |
| pslA | TGGCTACTACGACCTGCTGT | CAGGATGGTGATGTTGCGTT | 154 |
| algD | ACGCTGATCGACTTCGACAT | TGGTAGTTGCCGATGATGGT | 158 |
| katG | ATCGACGCTGCTGATGAAGA | CGGTAGATGTCGGTGATGGT | 147 |
| dnaK | GCTGGTGATGACGATGACGA | TCGATGGTGTCGTTGATGGT | 151 |
| rpoB | ATCGACGACGGTGATGAAGA | TGCTGATGTCGGTGTTGATG | 143 |
| 16S rRNA | AGAGTTTGATCCTGGCTCAG | TACGGCTACCTTGTTACGAC | 142 |

- Primer sequences are presented in the 5′–3′ orientation. Amplicon sizes were confirmed by agarose gel electrophoresis prior to quantitative analysis.

**Table S4**: **Relative gene-expression levels following treatment with selected compounds determined by qPCR analysis**

| **Gene** | **Control** | **Meropenem** | **5-Hydroxysophoranone** | **10′-Desmethoxystreptonigrin** | **Isoforsythiaside** |
| --- | --- | --- | --- | --- | --- |
| 16S rRNA | 1.00 ± 0.03ns | 1.01 ± 0.04ns | 0.99 ± 0.03ns | 1.00 ± 0.03ns | 0.98 ± 0.04ns |
| rpoB | 1.00 ± 0.04ns | 0.98 ± 0.03ns | 1.01 ± 0.04ns | 0.99 ± 0.03ns | 1.00 ± 0.04ns |
| pgm/algC | 1.00 ± 0.05 | 0.24 ± 0.02*** | 0.43 ± 0.03** | 0.29 ± 0.02*** | 0.56 ± 0.04* |
| hisIE | 1.00 ± 0.06 | 0.27 ± 0.02*** | 0.46 ± 0.03** | 0.31 ± 0.02*** | 0.61 ± 0.05* |
| ompA | 1.00 ± 0.05 | 0.35 ± 0.03*** | 0.54 ± 0.04** | 0.39 ± 0.03*** | 0.66 ± 0.04* |
| lasR | 1.00 ± 0.04 | 0.32 ± 0.02*** | 0.48 ± 0.03** | 0.37 ± 0.02*** | 0.59 ± 0.04* |
| pslA | 1.00 ± 0.05 | 0.29 ± 0.02*** | 0.45 ± 0.03** | 0.34 ± 0.02*** | 0.57 ± 0.04* |
| algD | 1.00 ± 0.05 | 0.31 ± 0.03*** | 0.49 ± 0.03** | 0.36 ± 0.02*** | 0.60 ± 0.04* |
| katG | 1.00 ± 0.04 | 0.41 ± 0.03** | 0.63 ± 0.04* | 0.47 ± 0.03** | 0.71 ± 0.05* |
| dnaK | 1.00 ± 0.04 | 0.44 ± 0.03** | 0.67 ± 0.05* | 0.49 ± 0.03** | 0.74 ± 0.05* |

- Values are presented as mean ± SD (n = 3) and normalized relative to untreated control samples using the 2−ΔΔCt method.
- Statistical significance was determined relative to untreated control groups. *p < 0.05; **p < 0.01; ***p < 0.001.
- pgm/algC: Phosphoglucomutase/ Phosphomannomutase; hisIE: Histidine biosynthesis enzyme; ompA: Outer membrane protein A; lasR: Quorum-sensing regulator; pslA: Polysaccharide biosynthesis protein; algD: Alginate biosynthesis protein; katG: Catalase-peroxidase; dnaK: Heat-shock chaperone protein.

**Table S5: Docking interaction profile and MD-retained contacts of selected compounds with PGM/AlgC and HisIE**

| **Target** | **Ligand** | **Binding energy (kcal/mol)** | **Main interacting amino acid residues from docking** | **No. of docking H-bonds** | **Hydrophobic / π / electrostatic interactions** | **H-bonds retained during MD** | **Major persistent contacts during MD** |
| --- | --- | --- | --- | --- | --- | --- | --- |
| PGM/AlgC | 5-Hydroxysophoranone | −7.961 | Arg15, His109, Asn110, Thr215, Thr306, Ser327, His329, Arg421, Ser423, Asn424, Thr425, Thr426, Lys285 | 2 | π–alkyl/alkyl contacts with Lys285; van der Waals contacts with Thr425, Thr426, Arg421, His329 | Not simulated | Not applicable |
| PGM/AlgC | 10′-Desmethoxystreptonigrin | −10.000 | Arg15, Asp18, His109, Lys118, Ser108, Thr215, Lys285, His329, Arg421, Ser423, Asn424, Thr425, Thr426 | 5 | π–alkyl and π–anion contacts involving Lys285/Asp18 region; van der Waals contacts with Thr425, Thr426, Ser423, Arg421 | 3 | Arg15, Ser108, Lys118, His329, Arg421 |
| PGM/AlgC | Isoforsythiaside | −7.137 | Arg15, Asp18, Ser108, His109, Lys118, Thr215, Lys285, His329, Arg421, Ser423, Asn424, Thr425 | 6 | π–alkyl contacts with Lys285; hydrophobic contacts around His329/Arg421 region; van der Waals contacts with Thr425 and Asn424 | Not simulated | Not applicable |
| PGM/AlgC | Meropenem | −6.682 | Arg15, Tyr17, Asp18, Ser108, His109, Lys118, His308, Asn424, Thr425, Thr426, Arg421, Ser423, Lys285, Glu375 | 2 | Electrostatic contacts with Asp18/Glu375 region; van der Waals contacts with Thr425, Thr426, Arg421 | Not simulated | Not applicable |
| HisIE | 5-Hydroxysophoranone | −8.800 | Met13, Leu34, Gly35, Tyr36, Phe53, Phe54, Ser55, Trp62, Lys64, Asp80, Asp82, Asp84, His96, Cys97, Cys104 | 3 | π–π/π–alkyl contacts with Phe53, Phe54, Tyr36, Trp62; hydrophobic contacts with Leu34 and Met13 | 2 | Asp80, Asp84, Lys64, His96 |
| HisIE | 10′-Desmethoxystreptonigrin | −7.961 | Met13, Leu34, Gly35, Tyr36, Phe53, Phe54, Ser55, Trp62, Lys64, Asp80, Asp82, Asp84, His96, Cys97, Cys104 | 4 | π–π/π–alkyl interactions with Phe53/Phe54/Trp62; hydrophobic contacts with Leu34 and Met13 | Not simulated | Not applicable |
| HisIE | Isoforsythiaside | −7.081 | Met13, Leu34, Gly35, Tyr36, Phe53, Phe54, Ser55, Trp62, Lys64, Asp80, Asp82, Asp84, His96, Cys97, Cys104 | 5 | π-associated contacts with aromatic residues; hydrophobic contacts with Leu34/Met13; van der Waals contacts with Gly35/Ser55 | Not simulated | Not applicable |
| HisIE | Meropenem | −6.812 | Met13, Leu34, Gly35, Tyr36, Phe53, Phe54, Ser55, Trp62, Lys64, Asp80, Asp82, Asp84, Thr96, Cys97, Cys104 | 2 | Electrostatic/hydrogen-bond contacts with Asp84/Thr96; hydrophobic contacts with Leu34 and Met13 | Not simulated | Not applicable |

**Supplementary Table S6. Active-site identification and AutoDock Vina grid-box parameters used for molecular docking**

| **Target** | **PDB ID** | **Chain** | **Selected docking region** | **Grid centre X, Y, Z (Å)** | **Grid-box dimensions (Å)** | **Exhaustiveness** | **Generated poses** |
| --- | --- | --- | --- | --- | --- | --- | --- |
| PGM/AlgC | 1K35 | A | Central catalytic cleft formed by residues from all four structural domains | 55.341, 51.701, 9.879 | 22 × 22 × 22 | 32 | ≥20 |
| HisIE | 6J2L | B | Conserved HisE-associated ligand-binding groove | 38.793, 34.217, 22.477 | 22 × 22 × 22 | 32 | ≥20 |

**Table S7: Integrated computational, antibacterial, enzyme-inhibitory, molecular-expression, and cytocompatibility profiles of the selected compounds**

| **Parameter** | **5-Hydroxysophoranone** | **10′-Desmethoxystreptonigrin** | **Isoforsythiaside** |
| --- | --- | --- | --- |
| **PGM/AlgC docking score (kcal/mol)** | −7.961 | **−10.000** | −7.137 |
| **HisIE docking score (kcal/mol)** | **−8.800** | −7.961 | −7.081 |
| **Overall docking profile** | Strong HisIE affinity and favorable PGM/AlgC affinity | Strongest PGM/AlgC affinity and favorable HisIE affinity | Moderate dual-target affinity |
| **Antibacterial activity, MIC range (µg/mL)** | 16–20 | **8–10** | 40.8–48 |
| **MBC range (µg/mL)** | 32–40 | **16–20** | 64–80 |
| **Selectivity index range** | 3.9–4.9 | **4.3–5.4** | 2.0–2.4 |
| **Cell-associated bacterial reduction (log10 CFU/mL)** | 2.68 | **3.51** | 2.06 |
| **PGM/AlgC IC50 (µM)** | 7.42 ± 0.38 | **3.21 ± 0.19** | 11.36 ± 0.64 |
| **HisIE IC50 (µM)** | 8.11 ± 0.41 | **3.88 ± 0.22** | 12.91 ± 0.72 |
| **Enzyme inhibition at 25 µM (%)** | 73.8 ± 2.6 | **86.4 ± 2.2** | 61.7 ± 2.9 |
| **PGM/AlgC protein expression** | 0.41 ± 0.03 | **0.28 ± 0.02** | 0.53 ± 0.04 |
| **HisIE protein expression** | 0.45 ± 0.03 | **0.31 ± 0.02** | 0.58 ± 0.05 |
| **OmpA protein expression** | 0.52 ± 0.04 | **0.43 ± 0.03** | 0.61 ± 0.05 |
| **LasR protein expression** | 0.49 ± 0.03 | **0.36 ± 0.02** | 0.57 ± 0.04 |
| **PslA protein expression** | 0.46 ± 0.03 | **0.34 ± 0.02** | 0.54 ± 0.03 |
| **Virulence/metabolic marker suppression** | Moderate suppression | **Strongest suppression** | Weak-to-moderate suppression |
| **A549 cell viability (%)** | 89.4 ± 3.2 | 76.3 ± 4.5 | **94.8 ± 2.6** |
| **BEAS-2B cell viability (%)** | 92.1 ± 2.8 | 81.7 ± 3.9 | **96.2 ± 2.3** |
| **CC50 (µM)** | 78.6 ± 4.1 | 42.8 ± 3.6 | **96.4 ± 5.2** |
| **Cytocompatibility profile** | High | Moderate | **Very high** |
| **Main strength** | Balanced dual-target activity with acceptable cytocompatibility | **Highest antibacterial, docking, enzyme-inhibitory, and molecular-suppression activity** | Best epithelial-cell compatibility |
| **Main limitation** | Lower antibacterial potency than 10′-Desmethoxystreptonigrin | Moderate cytotoxicity compared with the other natural compounds | Weaker antibacterial and enzyme-inhibitory activity |
| **Overall therapeutic profile** | Balanced activity–safety profile | **Most potent antibacterial lead, requiring toxicity optimization** | Safest scaffold, requiring potency optimization |

Values are summarized from the computational docking, antibacterial susceptibility, cell-associated antibacterial activity, enzyme inhibition, cytotoxicity, and Western blot analyses. Protein-expression values are normalized relative to untreated controls. Lower docking scores, MIC/MBC values, IC50 values, and protein-expression values indicate stronger activity, whereas higher cell viability and CC50 values indicate greater cytocompatibility.

**Table S8: Ligand library and reference compound used for molecular docking analysis**

| **Ligand** | **PubChem CID** | **Database/source** | **Compound type/class** | **Role in study** | **Selection rationale** |
| --- | --- | --- | --- | --- | --- |
| 5-Hydroxysophoranone | 42607927 | PubChem | Natural-product flavonoid-related scaffold | Screened phytocompound | Selected as a structurally diverse natural compound with hydroxyl-containing functional groups suitable for hydrogen-bonding interactions within bacterial enzyme pockets |
| 10′-Desmethoxystreptonigrin | 131994 | PubChem | Streptonigrin analogue / aminoquinone alkaloid | Screened phytocompound and lead antibacterial candidate | Selected because of its reported pharmacological relevance, microbial natural-product origin, quinone-containing scaffold, and predicted strong interaction with PGM/AlgC |
| Isoforsythiaside | 23958169 | PubChem | Glycosidic phenylethanoid/natural-product scaffold | Screened phytocompound | Selected as a glycosidic natural compound with multiple hydroxyl groups and comparatively favorable cytocompatibility profile |
| Meropenem | 441130 | PubChem | Carbapenem antibiotic | Reference antibacterial comparator | Included as a clinically used broad-spectrum antibacterial comparator for docking and experimental activity assessment |

The ligand library was a focused natural-compound set selected for dual-target screening against PGM/AlgC and HisIE. PubChem was used to retrieve two-dimensional structures and chemical identifiers. Ligands were converted into three-dimensional conformations, protonated at physiological pH, geometry-optimized using Avogadro software with the MMFF94 force field, and converted into PDBQT format using AutoDockTools 1.5.7/MGLTools before AutoDock Vina docking.

**Table S9: Molecular dynamics simulation parameters used for lead protein–ligand complexes**

| **Parameter** | **10′-Desmethoxystreptonigrin–PGM/AlgC** | **5-Hydroxysophoranone–HisIE** |
| --- | --- | --- |
| MD software | Desmond module, Schrödinger | Desmond module, Schrödinger |
| System preparation | System Builder module | System Builder module |
| Force field | OPLS4 | OPLS4 |
| Water model | TIP3P | TIP3P |
| Simulation box type | Orthorhombic | Orthorhombic |
| Box construction | System-specific box generated around the prepared protein–ligand complex | System-specific box generated around the prepared protein–ligand complex |
| Minimum buffer distance | 10 Å from solute to box boundary | 10 Å from solute to box boundary |
| Box dimensions | Automatically generated from solute dimensions using 10 Å buffer; fixed manual dimensions were not assigned | Automatically generated from solute dimensions using 10 Å buffer; fixed manual dimensions were not assigned |
| Neutralization | Counterions added | Counterions added |
| Salt concentration | 0.15 M NaCl | 0.15 M NaCl |
| Ensemble | NPT | NPT |
| Temperature | 300 K | 300 K |
| Pressure | 1 atm | 1 atm |
| Thermostat | Nosé–Hoover thermostat | Nosé–Hoover thermostat |
| Barostat | Martyna–Tobias–Klein barostat | Martyna–Tobias–Klein barostat |
| Equilibration protocol | Desmond default relaxation protocol: restrained minimization, restrained NVT/NPT equilibration, and final unrestrained equilibration | Desmond default relaxation protocol: restrained minimization, restrained NVT/NPT equilibration, and final unrestrained equilibration |
| Production simulation length | 500 ns | 500 ns |
| Trajectory recording interval | 100 ps | 100 ps |
| RMSD analysis | Protein backbone RMSD and ligand heavy-atom RMSD after receptor fitting | Protein backbone RMSD and ligand heavy-atom RMSD after receptor fitting |
| RMSF analysis | Residue-wise protein RMSF and ligand RMSF | Residue-wise protein RMSF and ligand RMSF |
| Protein–ligand interaction analysis | Hydrogen bonds, hydrophobic contacts, ionic interactions, water bridges, and contact frequency | Hydrogen bonds, hydrophobic contacts, ionic interactions, water bridges, and contact frequency |
| Structural compactness analysis | Radius of gyration | Radius of gyration |
| Solvent exposure analysis | Molecular surface area, solvent-accessible surface area, and polar surface area | Molecular surface area, solvent-accessible surface area, and polar surface area |
| Additional trajectory analysis | Secondary-structure elements and ligand torsional profiles | Secondary-structure elements and ligand torsional profiles |
| Binding-energy analysis | Prime MM/GBSA | Prime MM/GBSA |

**Supplementary Table S10: Computational parameters reported for reproducibility**

| **Computational step** | **Parameter** | **Reported value/detail** |
| --- | --- | --- |
| Receptor database | Source | RCSB Protein Data Bank |
| PGM/AlgC receptor | PDB ID | 1K35 |
| PGM/AlgC organism | Source organism | *Pseudomonas aeruginosa* |
| PGM/AlgC experimental method | Structure method | X-ray diffraction |
| PGM/AlgC resolution | Resolution | 2.20 Å |
| HisIE receptor | PDB ID | 6J2L |
| HisIE organism | Source organism | *Shigella flexneri* |
| HisIE experimental method | Structure method | X-ray diffraction |
| HisIE resolution | Resolution | 2.17 Å |
| Receptor preparation | Crystallographic water molecules | Non-essential water molecules removed |
| Receptor preparation | Polar hydrogens | Added |
| Receptor preparation | Kollman charges | Assigned |
| Receptor preparation | Non-polar hydrogens | Merged |
| Receptor preparation | Local geometry | Unfavorable local contacts corrected |
| Receptor minimization | Energy minimization | Performed before docking to improve local geometry and remove steric clashes |
| Receptor file conversion | Output format | PDBQT |
| Receptor conversion tool | Software/tool | AutoDockTools 1.5.7/MGLTools |
| Receptor inspection | Visualization tool | Discovery Studio Visualizer |
| Ligand database | Source | PubChem |
| Screened ligand 1 | Compound | 5-Hydroxysophoranone |
| Screened ligand 1 | PubChem CID | 42607927 |
| Screened ligand 2 | Compound | 10′-Desmethoxystreptonigrin |
| Screened ligand 2 | PubChem CID | 131994 |
| Screened ligand 3 | Compound | Isoforsythiaside |
| Screened ligand 3 | PubChem CID | 23958169 |
| Reference compound | Comparator | Meropenem |
| Reference compound | PubChem CID | 441130 |
| Ligand preparation | 2D-to-3D conversion | Performed before docking |
| Ligand preparation | Protonation state | Protonated at physiological pH |
| Ligand geometry optimization | Software | Avogadro |
| Ligand energy minimization | Force field | MMFF94 |
| Ligand conversion | Docking format | PDBQT |
| Ligand conversion tool | Software/tool | AutoDockTools 1.5.7/MGLTools |
| Active-site identification | Structural evidence | PDB records and published structural studies |
| Active-site prediction | Pocket-prediction tool | CASTp 3.0 |
| Active-site inspection | Visualization tool | Discovery Studio Visualizer |
| PGM/AlgC docking site | Selected binding region | Central catalytic cleft formed by residues from multiple domains |
| HisIE docking site | Selected binding region | Conserved putative ligand-binding groove within the HisE/HisI functional region |
| Docking software | Docking engine | AutoDock Vina |
| Docking search region | Grid placement | Restricted to identified catalytic/binding pockets |
| Docking search strategy | Whole-protein blind docking | Not performed |
| Docking grid values | Grid center and dimensions | Reported separately in Supplementary Table S6 from AutoDock Vina configuration files |
| Docking exhaustiveness | PGM/AlgC | 32 |
| Docking exhaustiveness | HisIE | 32 |
| Docking exhaustiveness rationale | Receptor-specific search depth | Higher value used for broader PGM/AlgC catalytic cleft; lower value used for more restricted HisIE binding groove |
| Docking output poses | Number of poses | At least 20 poses per ligand–target pair |
| Docking-pose selection | Selection criteria | Docking score, catalytic-pocket occupancy, hydrogen bonding, hydrophobic interactions, electrostatic complementarity, and absence of steric clashes |
| Interaction analysis | Software | Discovery Studio Visualizer |
| Interaction outputs | Recorded interactions | Interacting residues, hydrogen bonds, hydrophobic contacts, electrostatic contacts, π–π interactions, π–alkyl interactions, and van der Waals contacts |
| 2D interaction maps | Visualization | Discovery Studio Visualizer |
| 3D docking poses | Visualization | Discovery Studio Visualizer |
| Docking validation | Redocking feasibility | Not applicable because 1K35 and 6J2L do not contain suitable co-crystallized organic ligands |
| Docking validation alternative | Validation approach | Structure-guided pocket selection, CASTp prediction, conserved-residue inspection, restricted grid placement, interaction-profile analysis, and MD/MM-GBSA stability assessment |
| MD software | Simulation engine | Desmond module, Schrödinger |
| MD system preparation | Module | Desmond System Builder |
| MD force field | Force field | OPLS4 |
| Water model | Solvent model | TIP3P |
| Simulation box type | Box geometry | Orthorhombic |
| Simulation box construction | Solute–boundary distance | 10 Å buffer |
| Simulation box dimensions | Box dimensions | System-specific orthorhombic box automatically generated from solute dimensions using 10 Å buffer |
| Neutralization | Counterions | Added as required |
| Salt concentration | Ionic strength | 0.15 M NaCl |
| Simulation ensemble | Ensemble | NPT |
| Temperature | Simulation temperature | 300 K |
| Pressure | Simulation pressure | 1 atm |
| Equilibration protocol | Procedure | Desmond default relaxation/equilibration before production MD |
| Production simulation | Simulation length | 500 ns |
| Trajectory recording | Frame interval | 100 ps |
| Trajectory analysis | Stability parameters | RMSD, RMSF, secondary-structure analysis, protein–ligand contact mapping, radius of gyration, and SASA |
| Binding-energy analysis | Method | Prime MM/GBSA |
| MM/GBSA frame selection | Frames | Representative equilibrated trajectory frames |
| MM/GBSA energy terms | Components | Coulombic, hydrogen-bonding, lipophilic, covalent, solvation, and ligand-efficiency components |

## Figure S1: ****Molecular dynamics stability analysis of the 10′-Desmethoxystreptonigrin–PGM/AlgC complex.****

**(A)** RMSD profile of protein and ligand during simulation,

**(B)** Residue-wise RMSF analysis of the protein backbone,

**(C)** Secondary-structure element distribution,

**(D)** Secondary-structure timeline throughout the simulation trajectory,

**(E)** Protein–ligand contact frequency analysis,

**(F)** Time-dependent protein–ligand interaction mapping,

**(G)** Two-dimensional ligand–protein interaction diagram,

**(H)** Ligand RMSF profile,

**(I)** Ligand torsional-angle distribution analysis, and

**(J)** Ligand physicochemical property profiles including RMSD, radius of gyration, intramolecular hydrogen bonding, molecular surface area, solvent-accessible surface area, and polar surface area.

The simulation analyses indicated stable ligand accommodation and sustained protein–ligand interactions during the molecular dynamics trajectory.

## Figure S2: ****Molecular dynamics simulation analysis of the 5-Hydroxysophoranone–HisIE complex over a 500 ns trajectory****

**(A)** RMSD profile of protein and ligand during simulation,

**(B)** Residue-wise RMSF analysis of the protein backbone,

**(C)** Secondary-structure element distribution,

**(D)** Secondary-structure timeline throughout the simulation trajectory,

**(E)** Protein–ligand contact frequency analysis,

**(F)** Time-dependent protein–ligand interaction mapping,

**(G)** Two-dimensional ligand–protein interaction diagram,

**(H)** Ligand RMSF profile,

**(I)** Ligand torsional-angle distribution analysis, and

**(J)** Ligand physicochemical property profiles including RMSD, radius of gyration, intramolecular hydrogen bonding, molecular surface area, solvent-accessible surface area, and polar surface area.

The simulation analyses demonstrated stable conformational behavior and sustained interactions between 5-Hydroxysophoranone and the HisIE active site during the molecular dynamics trajectory.

## Figure S3: ****Representative triplicate Western blot images demonstrating reproducibility****

Three independent Western blot replicates showing PGM/AlgC, HisIE, OmpA, LasR, PslA, OprL, and GroEL expression under untreated control, Meropenem, 5-Hydroxysophoranone, 10′-Desmethoxystreptonigrin, and Isoforsythiaside treatment conditions. GroEL was used as the loading control.

**Figure S4: Representative agarose gel image showing qPCR amplification products of 16S rRNA, GroEL, rpoB, katG, dnaK, pslA, algC, algD, ompA, HisIE, and lasR in untreated control and compound-treated bacterial samples. Treatment groups included Meropenem, 5-Hydroxysophoranone, 10′-Desmethoxystreptonigrin, and Isoforsythiaside.**

## Figure S5: ****Western blot quantification and qPCR analysis of metabolic and virulence-associated markers following compound treatment.****

## **(A) Representative Western blot analysis of PGM/AlgC, HisIE, OmpA, LasR, PslA, OprL, and GroEL expression in bacterial cultures treated with Meropenem, 5-Hydroxysophoranone, 10′-Desmethoxystreptonigrin, and Isoforsythiaside compared with untreated controls. GroEL was used as the loading control.**

## **(B) Densitometric quantification of Western blot band intensities from three independent experiments. Protein-expression levels were normalized to GroEL and expressed relative to the untreated control group. Data are presented as mean ± SD; ns, not significant; *p < 0.05; **p < 0.01; ***p < 0.001.**

## **(C) qPCR expression profiles of 16S rRNA, rpoB, katG, dnaK, pslA, algC, algD, ompA, hisIE, and lasR following compound treatment.**

**Supplementary Figure S6: Three-dimensional binding poses of the selected compounds within the predicted catalytic or ligand-binding pockets of PGM/AlgC and HisIE.**

(A–D) Binding poses of 5-hydroxysophoranone, 10′-desmethoxystreptonigrin, isoforsythiaside, and meropenem within the PGM/AlgC catalytic cleft. (E–H) Corresponding binding poses within the HisIE ligand-binding groove. The receptors are shown as molecular surfaces or ribbon structures, the ligands are represented as sticks, and the principal interacting residues are labelled.
